# Medical data sharing and synthetic clinical data generation – maximizing biomedical resource utilization and minimizing participant re-identification risks

**DOI:** 10.1038/s41746-025-01935-1

**Published:** 2025-08-16

**Authors:** Simeone Marino, Ruth Cassidy, Joseph Nanni, Yuxuan Wang, Yipeng Liu, Mingyi Tang, Yuan Yuan, Toby Chen, Anik Sinha, Balaji Pandian, Ivo D. Dinov, Michael L. Burns

**Affiliations:** 1 Statistics Online Computational Resource (SOCR), University of Michigan, Ann Arbor, MI USA; 2 Department of Anesthesiology, University of Michigan Medical School, Ann Arbor, MI USA; 3 Department of Anesthesiology, Weill Cornell Medicine, New York, NY USA

**Keywords:** Health care, Data acquisition, Computational biology and bioinformatics, Computational models

## Abstract

The sensitive nature of electronic health records (EHR) and wearable data presents challenges in sharing biomedical resources while minimizing re-identification risks. This article introduces an end-to-end, titratable pipeline that generates privacy-preserving “digital twin” datasets from complex EHR and wearable-device records (Apple Watch data from 3029 participants) using DataSifter and Synthetic Data Vault (SDV) methods. Various obfuscation levels were applied (DataSifter: small, medium, large; SDV: CTGAN, Gaussian Copula) and benchmarked using utility (statistical fidelity, machine learning performance) and privacy (re-identification risk, detection likelihood) metrics. The highest-obfuscation DataSifter twin delivered the strongest privacy protection (0.83) while preserving key statistical and predictive signals (83.1% confidence interval overlap in regression models), outperforming SDV, particularly for longitudinal data. Despite declining performance in machine learning tasks with higher obfuscation, utility was generally preserved. The study underscores the importance of digital twin datasets and highlights DataSifter’s adaptability in privacy-utility trade-offs, advocating its utility for secure data sharing.

## Introduction

Translational science research has enormous promise for expediting clinical predictivity, optimizing resource utilization, and increasing the efficiency of instrument development and intervention dissemination to improve human health and wellbeing^1^. There remains a significant demand for clinical electronic health record (EHR) data, but due to the sensitivity of the data and privacy regulations, EHR data can be difficult to obtain and to use in research, model development and validation, and interdisciplinary projects. To mitigate this barrier, many groups have been working to develop solutions for the creation of realistic synthetic data: data that can be generated algorithmically, as digital twins modeled off of real datasets, and used in place of original datasets^2^. These synthetic data counterparts have applications across a wide variety of industries: they can be used to expand small social media datasets in order to feed more data to machine learning advertising model, as a benchmark for fraud detection, and in the case of medicine, can be used as a substitute for real EHR data that is locked away behind several layers of institutional and legal permissions. The latter application requires a great degree of precision in generating synthetic data, as this data will be used for important research projects and contains important protected health information.

Synthetic digital twin data generation is a nuanced process involving many different models and approaches. There are presently no effective mechanisms to enable sharing of, and collaboration with, big biomedical data without potential risks for either inappropriate release of sensitive information or a substantial degradation of the information content (data value and utility). Currently available protocols and algorithms for modeling, processing, interrogating, and ultimately sharing large sensitive data (e.g., millions of health records with thousands of heterogeneous features) include secure enclave access^3,4^, data encryption (e.g., fully homomorphic encryption-*fHE*)^5,6^ and *differential privacy* (*DP)*, a framework to protect individual data while allowing aggregate sharing of information)^7–15^. These all share a few advantages with many limitations^5,6,16–19^ and their practical use still lags behind research progress. For example, the appealing theoretical framework of DP, where a study dataset can be individually protected, but aggregate analysis can still be performed generated extensive academic research and an abundant supply of new algorithms^20^. Despite this, DP has not been adopted in practice, and existing applications are limited to specialized use cases^14^. Computational expense and lack of scalability represent the major bottleneck for DP-based technologies for most of the current methods. Recent efforts have tried to address these major limitations, but these methods are inherently not suitable for queries that return de-identified clones of the raw data^20^.

There exist many methods to generate digital twins^21–26^. To build a simple, reproducible, efficient, and effective demonstration of the utility of desensitized biomedical data, we selected two specific strategies, the Synthetic Data Vault (SDV) protocol^27^ and the *DataSifter* statistical obfuscator^28^ – to accomplish the following tasks: (i) generate realistic synthetic data (digital twin objects); (ii) evaluate the statistical properties of the desensitized digital twins; and (iii) quantify the balance between privacy (low-risk) and utility (high-information content) metrics. These methods provide a wide range of controls and support for joint distribution modeling of multiple variables and time-dynamic autocorrelations. Both the DataSifter and SDV methods mimic the joint distributions of mixed data types (numerical and categorical) and can handle numerical and categorical data. While DataSifter allows more control over different levels of de-identification, it may be computationally more efficient than SDV and may provide an improved approach for de-identification of longitudinal data. SDV has a limited “sequence” generation method that is designed for gene sequences and not appropriate to handle generic longitudinal data. We applied SDV and DataSifter on electronic health record (EHR) data and structured cross-sectional and consolidated longitudinal healthcare data based on a cohort of subjects wearing Apple Watch devices recording energy burnt and physical activity. The sensitive nature of these data demands efficient and secure de-identification and obfuscation techniques to consider data sharing.

The main contributions and novelty of this work include (1) the titratable digital twinning using the DataSifter, (2) creation of complex medical data, including longitudinal data, (3) use of wearable technology medical data, and (4) investigating a framework for how to evaluate synthetic data. In the spirit of transparent, reproducible, and grounded scientific discovery, we developed, validated, and shared an end-to-end pipeline ingesting complex datasets containing sensitive healthcare information and returning five digital twins derivative sets, each with different degrees of obfuscation. Each digital twin object is coupled with corresponding privacy, utility, and disclosure risk metrics that can be utilized by the data governors to decide on how the synthetic datasets can be shared. The information provided by our methodology informs stewards of sensitive biomedical data about likely disclosure risks associated with sharing synthetic digital twin healthcare data.

## Results

### Data

From 5459 participants, the following participants were excluded: those without wearable data (1868), missing weight and/or height (522), or only having AEB periods lasting >3 hours (40), yielding a final analytical (“original”) dataset of 3029 participants. Within this dataset, the mean (standard deviation) age was 45.4 (16.9) years, height was 169.3 (9.9) cm, weight was 83 (19.8) kg, and BMI was 28.8 (5.9). The majority were female (54.8%), married (58.4%), and Caucasian (50.6%). 18.3% identified as African American and 10.2% as Hispanic or Latino. Consolidation of ICD values reduced the unique patient-level ICD combinations from 2007 to 414. Summaries for the original and synthetic datasets can be found in Table 1.

The SDV package was used to synthesize two digital twin synthetic datasets (SDV_CTGAN and SDV_GC), while the DataSifter algorithm generated three digital twin synthetic datasets equating to three different levels of obfuscation (DS_Small, DS_Medium, and DS_Large). Computational times to generate the five clones are within 5 to 20 minutes, with larger levels of obfuscation taking longer times. DataSifter produced longitudinal data in <1 sec. Using the indirect PARSynthesizer method in SDV produced poor (flat) results, and this method was abandoned. For SDV data, AEB variables were synthesized directly as opposed to the synthesis of longitudinal data and calculation of AEB variables as was done in DataSifter. The SDV method was 10–20x slower than DataSifter. There is no metric for laboratory results as there were no aggregate variables such as AEB for wearable timeseries data. Because the SDV was unable to obfuscate laboratory data longitudinally, and we did not have a collapsible variable, the SDV could not be created, and metrics for comparison could not be performed.

**Table 1:** Study demographics

Variable	Level	Original	Data Sifter Small	Data Sifter Medium	Data Sifter Large	SDV GC	SDV CTGAN					
		Mean (SD)	Mean (SD)	Diff*	Mean (SD)	Diff*	Mean (SD)	Diff*	Mean (SD)	Diff*	Mean (SD)	Diff*
Age (years)	Age	45.4 (16.9)	45.5 (16.5)	0	45.3 (13.6)	−0.01	45.1 (7.5)	−0.02	45.2 (18.9)	−0.01	45.1 (18.4)	−0.02
Height (cm)	Height	169.3 (9.9)	169.2 (9.8)	0	169.2 (8.5)	−0.01	168.9 (4.8)	−0.04	169.4 (10.1)	0.01	166.3 (11.5)	−0.28
Weight (kg)	Weight	83 (19.8)	83 (19.5)	0	83.5 (16.1)	0.03	83.8 (8)	0.05	82.8 (19.7)	−0.01	80.4 (23.4)	−0.12
BMI	BMI	28.8 (5.9)	28.9 (5.8)	0.01	29.3 (6)	0.09	29.3 (2.1)	0.12	28.7 (5.7)	−0.01	29.3 (8.7)	0.06
		%	%	Diff*	%	Diff*	%	Diff*	%	Diff*	%	Diff*
Gender	Female	54.84	54.7	0	55.9	−0.02	65.7	−0.22	55.5	−0.01	52.9	0.04
	Male	45.16	45.3		44.1		34.3		44.5		47.1	
Married	Yes	58.4	58.4	0	61.7	−0.07	63.2	−0.1	59.6	−0.03	49.7	0.17
	No	41.6	41.6		38.3		36.8		40.4		50.3	
Race	Caucasian	50.6	50.6	0.01	50.6	0.05	59.0	0.21	52.2	0.04	36.0	0.39
	African American	18.3	18.4		18.2		18.2		17.0		21.8	
	Asian	19.3	19.4		20.6		15.7		18.8		17.9	
	Other	11.9	11.6		10.6		7.1		12.0		24.4	
Hispanic or Latino	No	89.8	90.2	−0.01	93	−0.11	98.4	−0.37	89.7	0	85.7	0.12
	Yes	10.2	9.8		7		1.6		10.3		14.3	
A	1	1.5	0.7	−0.07	1.3	−0.02	2.8	0.09	0.6	−0.09	0.7	−0.07
B	1	3.4	2	−0.09	2.5	−0.05	2.3	−0.06	2.6	−0.05	1.7	−0.11
C	1	5.3	2.6	−0.14	3.4	−0.09	5.2	−0.01	2.8	−0.13	1.9	−0.19
D	1	11.1	8.3	−0.09	10.2	−0.03	15.8	0.14	7.9	−0.11	7.5	−0.12
E	1	25.9	29.2	0.07	34.6	0.19	42.5	0.36	25.7	−0.01	20.9	−0.12
F	1	14.7	14.7	0	15.1	0.01	15	0.01	11.9	−0.08	9.7	−0.15
G	1	18.3	20	0.04	22	0.09	28.5	0.24	16	−0.06	13	−0.15
H	1	11.0	9.4	−0.05	10	−0.03	12.3	0.04	8.6	−0.08	6.9	−0.14
I	1	19.8	22.1	0.06	24.7	0.12	29.4	0.22	18.7	−0.03	15.9	−0.1
J	1	11.9	9.1	−0.09	9.2	−0.09	8.4	−0.12	6.7	−0.18	6.3	−0.19
K	1	16.8	17	0.01	18.9	0.05	21.4	0.12	12	−0.14	10.5	−0.18
L	1	9.4	7.6	−0.07	6.6	−0.1	6.2	−0.12	5.1	−0.17	4.9	−0.18
M	1	27.4	29.7	0.05	31.7	0.09	31.7	0.09	26	−0.03	18.6	−0.21
N	1	15.8	13	−0.08	13.3	−0.07	12	−0.11	10.7	−0.15	8.7	−0.22
O	1	3.6	3.1	−0.03	3.6	0	3.8	0.01	2.6	−0.06	2.1	−0.09
Q	1	1.5	0.3	−0.12	0.2	−0.14	0.1	−0.15	0.2	−0.15	0.4	−0.12
R	1	30.8	32.9	0.05	36.7	0.13	44.2	0.28	28.4	−0.05	22.4	−0.19
S	1	6.5	4	−0.11	4.8	−0.08	6.2	−0.01	4.9	−0.07	3.3	−0.15
T	1	3.0	0.2	−0.22	0.6	−0.19	0.9	−0.15	0.3	−0.22	0.1	−0.23
Z	1	33.9	36.3	0.05	41.2	0.15	49.1	0.31	33.9	0	23.7	−0.23
Any ICD	1	70.6	71.9	0.03	83.6	0.31	98.1	0.82	83	0.3	61.4	−0.19
Oth ICD	1		0.4		0.3		0		10.6		0.4	

*Standardized differences vs original data set.

### Univariate summaries

To investigate similarities of the synthetic data relative to the original, we used univariate comparisons for demographic and diagnosis group variables by calculating standardized differences between each synthetic dataset and the original dataset (Table 1). Medium absolute standardized differences were found in one variable for DS_Small (ICD T) and DS_Medium (Any ICD); eight variables in DS_Large (gender, race, Hispanic, and ICD E, G, I, R, Z); two in SDV_GC (ICD T and Any ICD); six times in SDV_CTGAN (height, race, and ICD M, N, T, and Z). A large absolute standardized difference was found only in Any ICD for DS_Large. All other absolute standardized differences were smaller than 0.2.

### Default outputs: Quality and diagnostic reports

We examined all privacy, utility, data integrity, and fidelity metrics generated using the SDV-functions available in the SDmetrics Python library. SDV privacy and utility metrics generation toolbox returns two main outputs by default from the SDMetrics library: a Quality Report and a Diagnostic Report. Table 2 illustrates both the Quality and Diagnostic Report table across the five digital twins. Due to the larger level of obfuscation, the DS_Large displays lower scores. However, the values are still high (e.g., > 0.8). The complete set of tables and scores used to generate the Quality and Diagnostic reports in Table 2 can be found in the **(**Supplementary Tables 1-4**)**.

**Table 2:** Quality and diagnostic reports for five levels of obfuscated datasets

Synthetic datasets	Quality*	Column Shape	Column Pair Trends
DS_Small	0.9644798	0.9659954	0.9629643
DS_Medium	0.9252010	0.9380709	0.9123311
DS_Large	0.8282870	0.8445582	0.8120159
SDV_GC	0.9168344	0.9472873	0.8863814
SDV_CTGAN	0.8597832	0.8832123	0.8363541

Synthetic datasets	Diagnostic**	Data Validity	Data Structure
DS_Small	0.9881699	0.9763398	1
DS_Medium	0.9929982	0.9859965	1
DS_Large	0.9990784	0.9981567	1
SDV_GC	0.9929019	0.9858039	1
SDV_CTGAN	0.9838781	0.9677561	1

*: Quality column averages the Column Shape and Column Pair Trends values representing column shapes and correlations. **: Diagnostic column averages the Data Validity and Data Structure values. The score is normalized between 0 and 1, where 0 is usually not good, and 1 is best. See Supplementary Information for a detailed breakdown of both Quality and Diagnostic reports metrics.

**Fig. 1:**
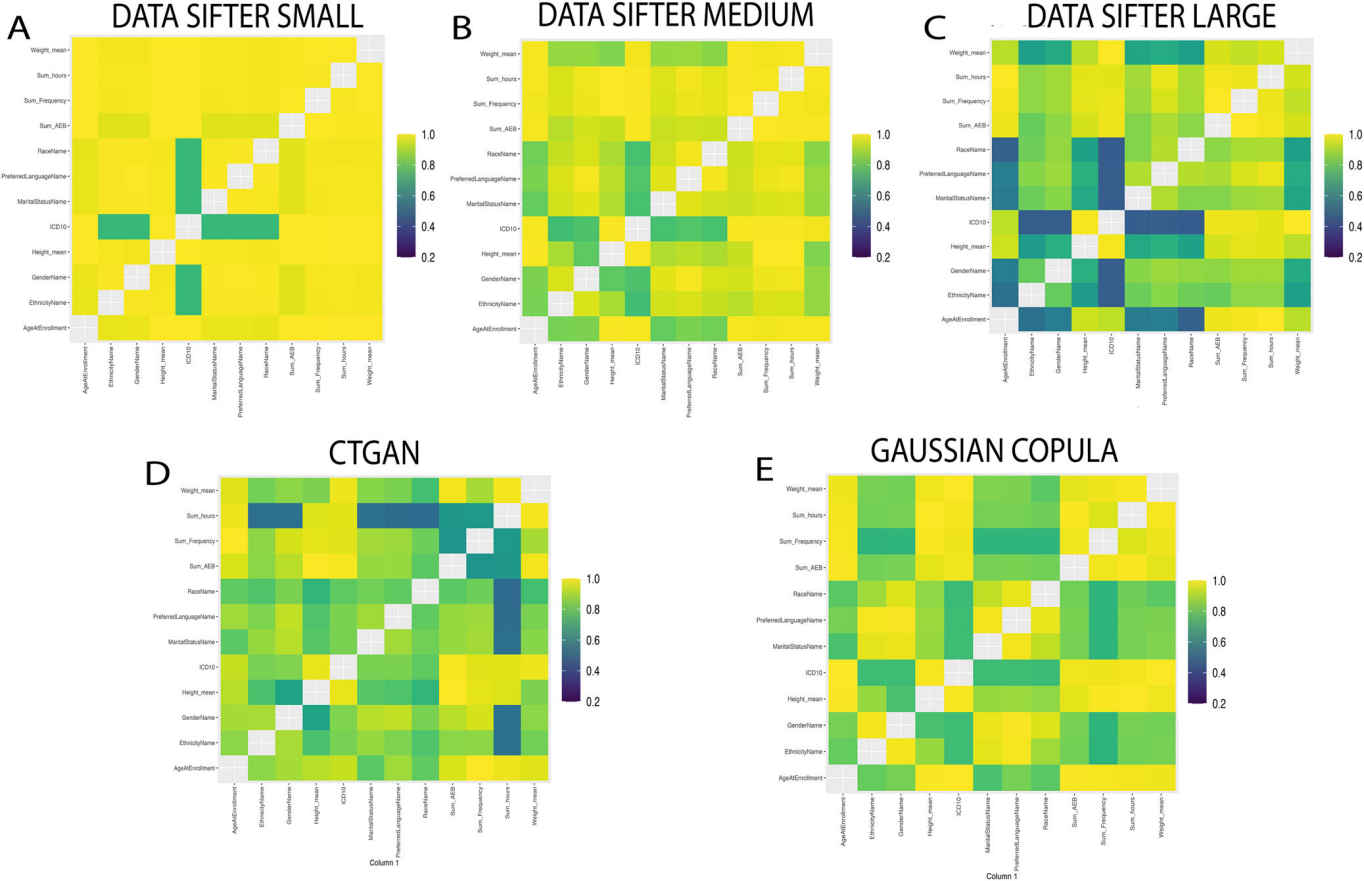
Heatmap representation of the Column Pair Trends metrics applied to the five synthetic datasets. The Diagnostic Report is shown in Supplementary Information and comprises a set of metrics for data fidelity, integrity, and structural congruence. Both DataSifter and SDV-based obfuscation generated digital twins with a score of 1 (optimal). The next sections will illustrate a larger set of metrics that we label as either utility or privacy metrics. **A** DataSifter_Small, **B** DataSifter_Medium, **C** DataSifter_Large, **D** CTGAN, and **E** Gaussian Copula.

An intuitive way to showcase the results and identify differences is to display the correlation structure with heatmaps. Figure 1 shows different heatmaps for each digital twin and breaks down the impact of each variable to each metric and ultimately to the overall score. Overall, the heatmaps in Fig. 1 revealed a consistent correlation structure across the Original and DataSifter_Small datasets, while larger obfuscation levels led to reduced homogeneity. Both DataSifter and SDV obfuscation highlight how the numerical variable ICD10 is the most affected by the obfuscation process (lowest scores overall). The heatmaps should also be used to evaluate how the obfuscation method is affecting the whole dataset homogeneously or not. For example, the higher impact on ICD10 is uniquely emerging from the CTGAN method in Fig. 1 (i.e., darker row/column associated to ICD10), whereas the DataSifter_Large shows a more homogeneous obfuscation across the entire dataset. Unless a specific outcome is targeted for obfuscation, having an homogeneous display of dissimilarity is recommended. Supplementary Information also displays scores for the Overall Data Validity and Data Structure for both categorical (Supplementary Table 5**)** and numerical (Supplementary Table 6) variables.

### Overall privacy and utility scores

We consolidated the many privacy and utility metrics scores under a single score wherever feasible (e.g., similar ranges) and meaningful (e.g., similar interpretation). Table 3 shows the overall utility and privacy scores across the five obfuscated datasets as compared to the Original dataset. The scores show similar performances when comparing DataSifter to SDV. The overall privacy scores are only computed for privacy metrics that do not need to specify the variable types and are global measures. Supplementary Information shows more privacy scores intended to quantify the risk of inference against a hacker attack towards categorical and/or numerical sensitive variables **(**Supplementary Tables 7–13**)**.

**Table 3:** Overall privacy and utility scores

Synthetic datasets	Overall Privacy Scores	Overall Utility Scores (Categorical targets)		
		Gender Name	Marital Status Name	Ethnicity Name
DS_Small	0.5456944	0.8624495	0.6724815	0.5920355
DS_Medium	0.6711206	0.6673633	0.4907246	0.2768268
DS_Large	0.8312806	0.6207404	0.3706400	0.2383583
SDV_GC	0.6736377	0.7703573	0.3988791	0.2662551
SDV_CTGAN	0.8296063	0.7258304	0.4217817	0.2397493

Overall Privacy Scores is calculated for privacy metrics that do not need to specify the variable types. The following table calculates the average score of *NewRowSynthesis* (Supplementary Information), *LogisticsDetection* (Supplementary Table 11), *SVCDetection* (Supplementary Table 11), and *TableStructure* (Supplementary Information). Example of Overall Utility Scores for three different categorical features. The scores range from 0 (Worst: The real data is not at all safe from the attack. The attacker can correctly guess every sensitive value by applying the chosen attack algorithm.) to 1 (Best: the real data is 100% safe from the attack. The attacker is not able to correctly guess any of the sensitive values by applying the chosen attack algorithm).

Utility scores assume a target variable to be predicted. When the variable is binary, categorical, or Boolean, the overall utility score is the average score of BinaryAdaBoostClassifier, BinaryDecisionTreeClassifier, BinaryLogisticRegression, and BinaryMLPClassifier (Supplementary Table 14). For multiclass categorical or Boolean variables, the metric is the average score of MulticlassDecisionTreeClassifier, MulticlassMLPClassifier (Supplementary Tables 15, 16). Finally, to ensure compliance with the overall metric range, from 0 to 1.0, the only utility score for a numerical target is given by the LinearRegression metric (Supplementary Table 17). The target features for the average utility scores in Table 3 are all categorical. Supplementary Information also shows another ensemble of Privacy/Utility metrics, namely the Categorical and Numerical Privacy Against inference scores **(**Supplementary Tables 18, 19**)**.

### Utility metrics: Generalized Linear Models results comparisons

**Fig. 2:**
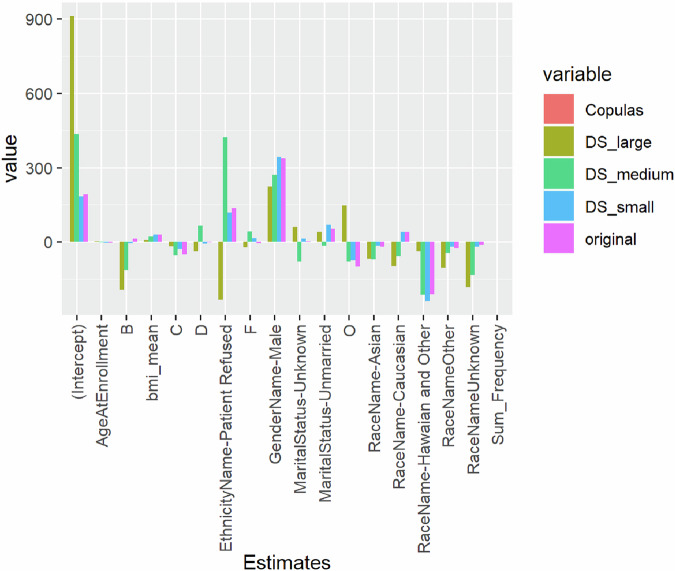
Multivariable model results. After fitting linear regression models for the original and each synthetic dataset, we calculated the percentage of confidence interval overlap for each explanatory variable estimate (Supplementary Table 20). The model fit on the DataSifter_Large dataset appeared to have confidence interval estimates most consistent with that of the original, ranging from 51.7% to 96.8% and with an average percent overlap of 83.1%. The models fit on the DataSifter_Medium and Small datasets had medium consistency with that of the original, with average percent overlaps of 63.7% and 63.1%, respectively. Lastly, the models fit on the SDV-copula and SDV-CTGAN datasets had the poorest consistency with that of the original, with average percent overlaps of approximately 50% for both.

In Fig. 2 we display results from a Generalized Linear Model (GLM) as bar plot estimates deviating from zero. The GLM was created with AVG_AEB_day (calculated as Sum_AEB/Sum_hours/24) as the response/output with thirty-eight covariates, including BMI, age, gender, marital status, race, ethnicity, preferred language, and others. The variable ICD10 is represented as twenty binary variables corresponding to the first letter of the ICD10 code. In Fig. 2, we display only the GLM estimates for significant covariates in the GLM model across the original dataset and the five different obfuscated datasets. The results for the SDV_CTGAN are omitted because all the estimates were not significantly different from zero, illustrating how the CTGAN dataset dramatically lacks utility when predicting AVG_AEB_day. Overall, there is a consistency across the different obfuscated datasets in identifying the same subset of significant covariates, both across the obfuscated datasets and in comparison, with the original dataset (purple bars). Moreover, the sign of the parameter estimates matches the original dataset estimates. Unsurprisingly, the largest difference is in comparison to the DS_Large dataset (i.e., military green bars) estimates. The complete set of results is available in the Supplementary Information.

We additionally calculated standardized differences for each regression point estimate, to compare each estimate from the model fit on each synthetic dataset to that of estimates fit on the original analytical dataset **(**Supplementary Table 20**)**. Across all standard deviations, the DataSifter_Large dataset exhibited the smallest median standard difference (0.51), followed by DataSifter_Medium (0.69). The DataSifter_Small dataset had a median standard difference of 1.4. The SDV-copula and SDV-CTGAN datasets had very large median standardized differences, 1766 and 22795, respectively.

## Discussion

There exist no effective mechanisms to rapidly enable broad sharing of and collaboration with large healthcare data without risking either inappropriate release of sensitive information or potential degradation of the information content. Protocols and algorithms such as secure enclave access^3,4^, data encryption^5,6^ and differential privacy^9^ all share a few advantages with many limitations^15–19^, and their practical use still lags behind research progress^14,29^. Our study demonstrates that synthetic data generation, particularly through the DataSifter framework and digital twinning, effectively balances privacy protection and data utility preservation of sensitive biomedical datasets. These findings support the development of synthetic healthcare datasets and highlight the tradeoff between maintaining utility (identifying and providing clinical insight) and privacy. Given this tradeoff, synthetic data offers improved accessibility by allowing broader data sharing while protecting sensitive information, but comes at the potential expense of data utility, as increased obfuscation may reduce the accuracy and reliability of analyses.

Our approach in generating privacy (disclosure) and utility metrics is similar in scope and results to related studies and similarly includes the use of GAN, multiple imputation, correlation structure, support coverage, and attribute disclosure^2,30^. Our study differentiates by using healthcare data with increased complexity, including time-series, categorical, and continuous variables. As healthcare data is often incomplete and varied, feature engineering was still required to achieve the level of synthetic data quality we achieved. Specifically, univariate summaries correlated with level of obfuscation with eight variables attaining medium absolute standardized differences and one large relative to the original data in DS_Large. DS_Medium and DS_Small each with one variable, while SDV_GC had two and SDV_CTGAN had six. Quality and diagnostic reports also correlate with obfuscation, though all synthetic data sets performed well (values > 0.8). Overall privacy scores again correlate, with DS_Large displaying the highest (0.83) but lowest in overall utility (0.23−0.62). Generalized linear models created from original and synthetic datasets using AEB as the output showed DS models greatly outperforming SDV in both correlation to significant covariates and standardized differences for each regressive point estimate. This study makes four specific contributions.

First, it delivers a tunable DataSifter framework that provides data governors with full control over privacy by choosing different levels of obfuscation, thereby creating “digital twins” suited to diverse risk profiles. Second, it demonstrates the first integrated synthesis of heterogeneous structured EHR, laboratory, and longitudinal wearable streams, expanding the scope of realistic biomedical twins. Third, it incorporates data from wearable technology. Fourth, every released twin is bundled with an evidence-based battery of utility (statistical fidelity, ML performance) and privacy (re-identification, inference-attack) metrics, empowering transparent data-sharing decisions to evaluate synthetic data. In our head-to-head evaluations, we found DataSifter to be both faster and more protective than a state-of-the-art synthetic data vault. The DataSifter maintains moderate utility, establishing a superior blueprint for secure, high-quality dissemination of sensitive biomedical data and fostering reproducible translational research

Overall, DataSifter and DLSO methods of digital twinning performed better and may allow more robust development of synthetic healthcare data creation and use in clinical research. By introducing adjustable obfuscation levels, DataSifter enables data governors to tailor synthetic outputs to specific privacy requirements without catastrophic loss of analytical value, providing both the ability to control the level of obfuscation induced on the sensitive data and an efficient and effective way to address obfuscation of more complex healthcare data, specifically longitudinal data. In contrast, traditional methods of obfuscation, such as SDV methods, produced synthetic data with poor longitudinal fidelity, higher vulnerability to inference attacks, and low statistical coherence (confidence interval overlap for key covariates). While hyperparameter optimization was not the focus of this work, it would be beneficial to better understand the performance and the relation to method feature decisions. In future work, we will explore Bayesian optimization approaches to investigate the hyperparameter space and improve ML performances with respect to the utility metrics generation on different sensitive target outcomes.

There are several limitations that should be highlighted in this work. (1) This specific study focused on a single cohort from a single institution, using structured EHR and wearable data. Future work should validate these methods on unstructured or multi-modal datasets from multi-institutional datasets and employ model-free AI prediction and unsupervised ML techniques to evaluate the balance between privacy protection and utility preservation of the digital twin clones. (2) There remain additional complexities of healthcare data beyond the use of longitudinal data, which were not addressed in this study. (3) Computational inefficiencies in SDV’s longitudinal synthesis warrant algorithmic refinements, which may be available but unrecognized by this study team. (4) In this manuscript, we do not comment on privacy laws of specific regions and geographies but maintain that preventing re-identification of patients is paramount to medical research. In the EU and the US, the General Data Protection Regulation (GDPR) and the Health Insurance Portability and Accountability Act (HIPAA), respectively, protect patient information. Synthetic data can still be under the authority of these privacy laws if any risk exists that can link synthetic data back to individuals. The techniques discussed in the paper are novel methods to minimize that risk as much as possible, but further work is needed to understand how sensitive and derived synthetic data can safely be shared. (5) Data processing included ICD categorical groupings recast as integers due to categorical level limitations in DataSifter. Such a recasting can introduce bias and possible incongruent mapping between the original and synthetic ICD10 labels. Furthermore, this preprocessing reduced data granularity, limiting downstream uses such as differentiating subclasses of ICD codes. Despite these challenges, the framework provides a reproducible pathway for secure data sharing, fostering collaborative innovation in translational research. By prioritizing both privacy and utility, synthetic data generation emerges as a critical tool for advancing biomedical discovery in an era of heightened data sensitivity.

## Methods

### Data

In this study, we used a retrospective participant cohort combining EHR records with wearable data collected from participants between Aug 14, 2018, and Dec 19, 2019. Wearable data was collected using Apple Watch Series 3 or 4 (Apple, Cupertino, CA, USA), an Omron Evolv Wireless Blood Pressure Monitor (Omron, Kyoto, Japan), and the MyDataHelps study smartphone application (CareEvolution, Ann Arbor, MI, USA), as described previously^29,31^. All data was extracted from the University of Michigan and was reviewed and approved by the University of Michigan’s Institutional Review Board (HUM00221930). The authors followed STROBE guidelines^32^. All data were linked using de-identified participant IDs and ordered by date and time of observation. EHR data consisted of laboratory data (laboratory name, result, units of measure, and normal range), 10th revision of the International Classification of Diseases (ICD), and patient demographics (age at enrollment, gender, marital status, race, ethnicity, preferred language, height, weight, and BMI). Longitudinal wearable data consisted of device information (model, manufacturer, and software/hardware version), active energy burned (AEB, start date/time, end date/time, kcal burned). Participants were excluded if they did not have wearable data or demographic data. Additionally, AEB events greater than 3 hours in continuous duration were excluded as these events were found to be device recording errors upon manual review.

### Data processing

Variables were processed prior to synthetic data creation as follows: laboratory values were determined to be high, low, or within normal range based on laboratory ranges provided in the data. ICD values were grouped according to their parent alpha value, for example “I25.10” -> “I.” Parent alpha values were consolidated, excluding categories with low representation (removing “W”, “U”, “V”, “Y”, “P”, and “X” ICD-10 categories). ICD alpha combinations were further reduced by consolidating ICD combinations with a frequency of one (found in only one patient) into the closest overlapping non-single combination. Height, weight, and BMI means were averaged by patient whenever multiple results existed. For the purposes of the obfuscation algorithms, remaining ICD combinations were recast as categorical integers and then binarized across the remaining twenty letters comprising each ICD code.

For synthetic data, height and weight were synthesized and BMI was derived from synthetic weight and height. We used a single outcome for multivariable modeling for synthetic and original data, daily-normalized active energy burned (AEB). AEB was selected from the available longitudinal wearable data as a realistic variable for modeling. While there are potential correlations to patient demographics, the goal is to use multivariable models to evaluate synthetic data relative to the original. To calculate the outcome of AEB per patient, we first summed AEB across all events recorded for the patient to obtain the total AEB. Then, we totaled the seconds recorded for each event of active energy burned (AEB end - AEB start) to obtain the total seconds active for each patient. This was then converted to days, dividing by (60 sec/min * 60 min/hr * 24 h/day). We then averaged this AEB rate for each patient by dividing the total AEB by the total days active, to calculate the outcome of AEB. Both AEB and mean BMI were omitted from the DataSifter obfuscation step since they are fully dependent on other features and calculated after obfuscation

### Synthetic data creation - DataSifter

The DataSifter algorithm was used to create synthetic digital twin data simulated from the joint distribution of the observed non-longitudinal data using R performed on an AMD Ryzen 9 workstation (code shared in this repository https://github.com/SOCR/DataSifter) as described previously^33–35^. Two core iterative processes support the DataSifter statistical obfuscation: imputation and obfuscation. More information on DataSifter can be found in Supplementary Information. The general DataSifter obfuscator allows continuous level of desensitization from none (raw data) to complete (synthetic digital twin data). In this study, we used DataSifter with three categorical levels of obfuscation including “small,” “medium” and “large.” Within each obfuscation level, we controlled sifting parameters, such as (1) proportion of artificially introduced missingness, (2) number of repeated paired iterations of artificial missingness and imputation, (3) proportion of obfuscated features (numerical or categorical), and (4) swapping proportion of closest neighboring cases. For longitudinal (time series) data, the DataSifter Longitudinal Obfuscator (DSLO) was used (Supplementary Fig. 1-4). Like DataSifter, DSLO generates three different levels of obfuscation (small, medium, and large).

### Synthetic data creation - Synthetic Data Vault

The Synthetic Data Vault (SDV) is a free python library originally developed at MIT^27^. SDV is designed for generating synthetic data that mimics real datasets while preserving their statistical properties and relationships. It offers various models and methods, including tabular, relational, and time-series data synthesis. SDV is designed for scenarios where data privacy is crucial, as it allows users to create realistic synthetic datasets for analysis, machine learning, or sharing without exposing sensitive information. SDV (version 0.17.2) was used to generate a single level of synthetic data for the original dataset, including longitudinal data via joint probability distributions using Gaussian copula and a *Conditional Tabular Generative Adversarial Network* (CTGAN) (Supplementary Fig. 5-6). The SDV package generated two digital twin synthetic datasets, named SDV_CTGAN and SDV_GC (Gaussian Copula). There is no direct method to generate synthetic longitudinal data in the SDV package. The PARSynthesizer is the only option, and it uses a deep learning method to train a model for generating synthetic sequence data (e.g., gene sequences).

### Synthetic data evaluations

SDMetrics is a companion library to SDV and provides a range of metrics to assess how well synthetic data matches the original data in terms of distribution, correlations, and other statistical properties. We used SDMetrics (version 0.13.1) to quantitatively measure the effectiveness of synthetic data generation through privacy and utility metrics across each digital twin (obfuscated) dataset, as compared to the original data. As SDMetrics generates a large set of privacy and utility metrics, wherever feasible, we consolidate privacy or utility metrics under the same overall scores. It is worth noting that the interpretation of a score can be subjective, whether the data governor prioritizes privacy versus utility. A high score for privacy may be associated with lower utility, and vice versa. A detailed list and description of evaluation metrics is described in Supplementary Information, with equations from less common mathematical formulas used in these evaluations.

Univariate summaries for variables were compared across all data sets. Categorical variables were summarized using proportions and frequencies. Means and standardized differences were used for summarizing approximately normal interval variables and medians and quartiles were used to summarize non-normal interval variables. Standardized differences for each variable were used to compare each synthetic dataset to the original dataset. Absolute standardized differences greater than 0.8 were considered large, 0.5−0.8 were considered medium, 0.2−0.5 were considered small, and those smaller than 0.2 were considered negligible.

Utility and privacy metrics were used to evaluate synthetic data results relative to the original dataset. Utility metrics returned scores ranging from 0.0 (Worst: Given the training data with the provided ML algorithm, you will not be able to predict any of the test data correctly) to 1.0 (Best: Given the training data with the provided ML algorithm, you will be able to perform ML tasks with 100% accuracy on the test data). Privacy metrics measure the risk of disclosing sensitive information through an inference attack and describe how difficult it is for an attacker to correctly guess sensitive (original) information and risk of an attacker being able to infer original values from a digital twin. We used several ML algorithms for different sensitive target classes for privacy evaluations. Privacy scores range from 0.0 (Worst: suggesting the real data may be at high risk for re-identification or machine learning model can perfectly identify synthetic data apart from the real data) to 1.0 (Best: the optimal protection of the real data against potential re-identification or the machine learning model cannot identify the synthetic data apart from the real data). In this case, an attacker is not expected to correctly match any of the sensitive information in the digital twin data objects, and hence participant information is strongly protected against re-identification

### Multivariable modeling

We used a supervised machine learning approach to assess the performance of the alternative data obfuscators. Specifically, for the original dataset and each of its synthetic digital twins, we fit linear regression models to predict the same clinical *outcome*, the daily-normalized active energy burned, based on all other EHR data elements. The results of these linear models facilitate the assessment of how well synthetic digital twins preserved key associational patterns relevant to health outcomes. Explanatory variables included age, BMI, gender, marital status, race, and comorbidity diagnoses. Multivariable results were compared by examining the overlap in the confidence intervals and standardized differences corresponding to each variable.

## Data Availability

The datasets generated and/or analyzed during the current study are not publicly available as they are defined as limited datasets per United States Federal Regulations and require execution of a data use agreement for transfer or use of the data. The investigative team is able to share data securely and transparently conditional on: (i) receipt of a detailed written request identifying the requestor, purpose and proposed use of the shared data, (ii) use of a secure enclave for the sharing of personally identifiable information and (iii) the request is permissible within the confines of existing data use agreements.

## g

Supplementary Information
