# Supplementary material for: Medical data sharing and synthetic clinical data generation – maximizing biomedical resource utilization and minimizing participant re-identification risks
Source: NPJ Digit Med. 2025 Aug 16;8:526. doi: 10.1038/s41746-025-01935-1 (PMC12357896; doi:10.1038/s41746-025-01935-1)
Supplement: Supplementary file 1 — Supplementary Information [file 41746_2025_1935_MOESM1_ESM.pdf]

Supplementary Information

Summary	Topic	Page
Supplementary Note 1	DataSifter	2
Supplementary Note 2	DataSifter Longitudinal data Obfuscator (DSLO)	4
Supplementary Note 3	Synthetic Data Vault (SDV)	9
Supplementary Note 4	Data Evaluation Metrics	13

Supplementary Note 1 - DataSifter

The DataSifter technique provides data-governors with control over the tradeoffs of data utility and privacy protection. Iterative data processing includes stochastic identification and manipulation of candidate elements, which results in a synthetically generated sifted dataset. This statistical obfuscation modifies cases (e.g., subjects, participants, units) and features (e.g., variables or data elements) by selecting, nullifying, and iteratively imputing the data. This process heavily relies on statistical multivariate imputation to preserve the joint distributions of the complex structured data archive. At each step, the algorithm generates a complete dataset that in aggregate closely resembles the intrinsic characteristics of the original cohort, however, on an individual level (e.g., rows), the data are substantially obfuscated. The risks for subject re-identification by stratification using the sifted output is reduced, as meta-data for all subjects is repeatedly and loosely encoded.

A number of techniques including mathematical modeling, statistical inference, probabilistic (re)sampling, and imputation methods are embedded in the DataSifter information obfuscation protocol. As data stewards can keep a mapping between the native subject identifiers (e.g., electronic health record, EHR) and the study-specific subject IDs (sequential or random), the size and complexity of the data collection may easily be extended to add additional longitudinal data augmenting previously generated DataSifter output datasets. This provides a meaningful mechanism for aggregation of obfuscated data.

In detail, first, the DataSifter algorithm randomly and artificially generates missingness in the data, following a Missing Completely At Random (MCAR) structure,[1] and uses robust multivariate imputation methods, e.g., MissForest,[2] to approximate the original information. Second, DataSifter classifies neighboring cases using Euclidean and Gower distances for

continuous and categorical variables, respectively. Within each neighborhood cluster, the DataSifter randomly swaps a subset of feature values between similar records. The second operation guarantees partial change for each record while preserving the joint distribution and geometrical information on the data in feature space.[3–5]

DataSifter parameters can be tuned to generate different desensitization results. Our previously published work was used to validate preset parametrizations and label synthetic outputs as “small,” “medium” and “large.” We have two parameters related directly to artificially introduced missingness impact on synthetic data generation, namely the proportion of artificial missingness (k1) and how many times the cycle missingness and imputation is performed (k2). The level “small” corresponds to k1=5% and k2=1. The level “medium” corresponds to k1=25% and k2=2. The level “large” corresponds to k1=40% and k2=5. We are working on providing a mathematical framework for the DataSifter to investigate convergence and statistical properties, as well as assess the optimal set of parameters to appropriately map and differentiate levels of desensitization (i.e., small, medium, and large). Ultimately these labels are contingent to the data under analysis. That is why incorporating privacy and utility metrics as a post-optimization step can validate these choices.

Supplementary Note 2 - DataSifter Longitudinal Data Obfuscator (DSLO)

The DataSifter Longitudinal Data Obfuscator (DSLO) expands on the DataSifter briefly described in **Supplementary Note 1** to address longitudinal data obfuscation. DSLO first addresses missingness via the Seasonal-Trend decomposition using Loess (STL) method for data interpolation (**Supplementary Figure 1**).[6,7]

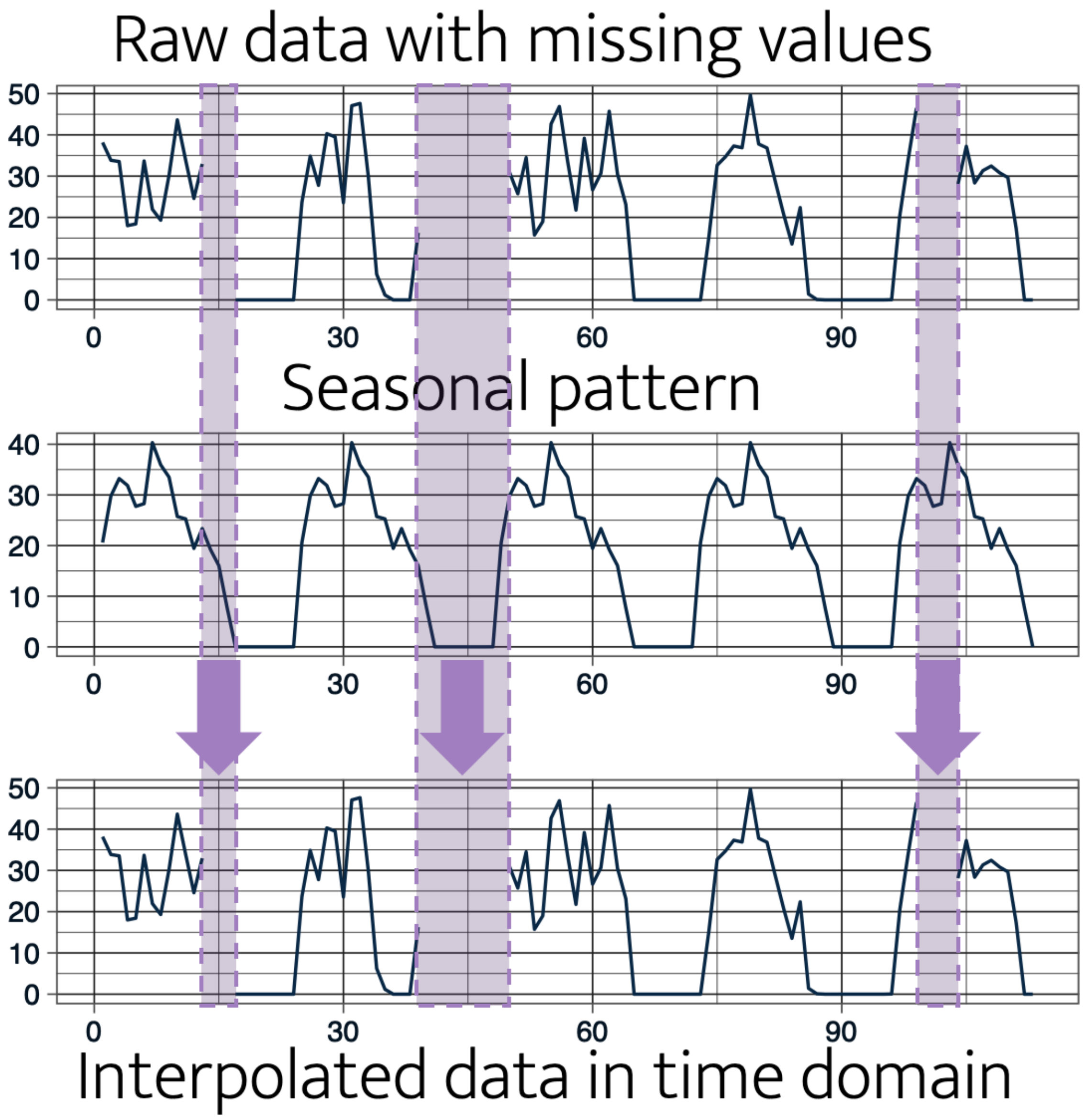

Supplementary Figure 1: DataSifter Longitudinal Obfuscation - data incompleteness.

DSLO first addresses missingness via the Seasonal-Trend decomposition using the Loess (STL) method for data interpolation.

Fourier Transformation into Frequency Domain

The interpolated time series, with its length denoted by l, is transformed into the frequency domain by the Fast Fourier Transform (FFT),[8] encompassing frequencies up to the maximum value of l. This frequency domain transformation is a crucial precursor to the implementation of subsequent data obfuscation techniques.

Data Obfuscation

Data obfuscation comprises an initial filtering step. Within the frequency domain, a filtering process is applied to the time series using a parameter known as depth, d, which determines the proportion of signals to be protected, with a lower depth value corresponding to a diminished obfuscation level. To obfuscate the unprotected signals in high frequencies, both the real and imaginary parts were resampled utilizing kernel density estimation with a Gaussian kernel.[9] The bandwidth of the Gaussian kernel was determined by $k \times \left(\frac{4\hat{\sigma}^2}{3n}\right)^{\frac{1}{5}}$, where k is a factor indicating the smoothness of the kernel (e.g., a larger k implies a higher smoothness and, consequently, a higher level of obfuscation), $n = (1 - d)l$ represents the length of the unprotected signals and $\hat{\sigma}$ is the standard deviation of the unprotected signals of the real and imaginary parts respectively (see **Supplementary Figure 2**). **Supplementary Figure 3** illustrates the differences among d settings for three levels of obfuscation — low (green), medium (blue), and high (red).

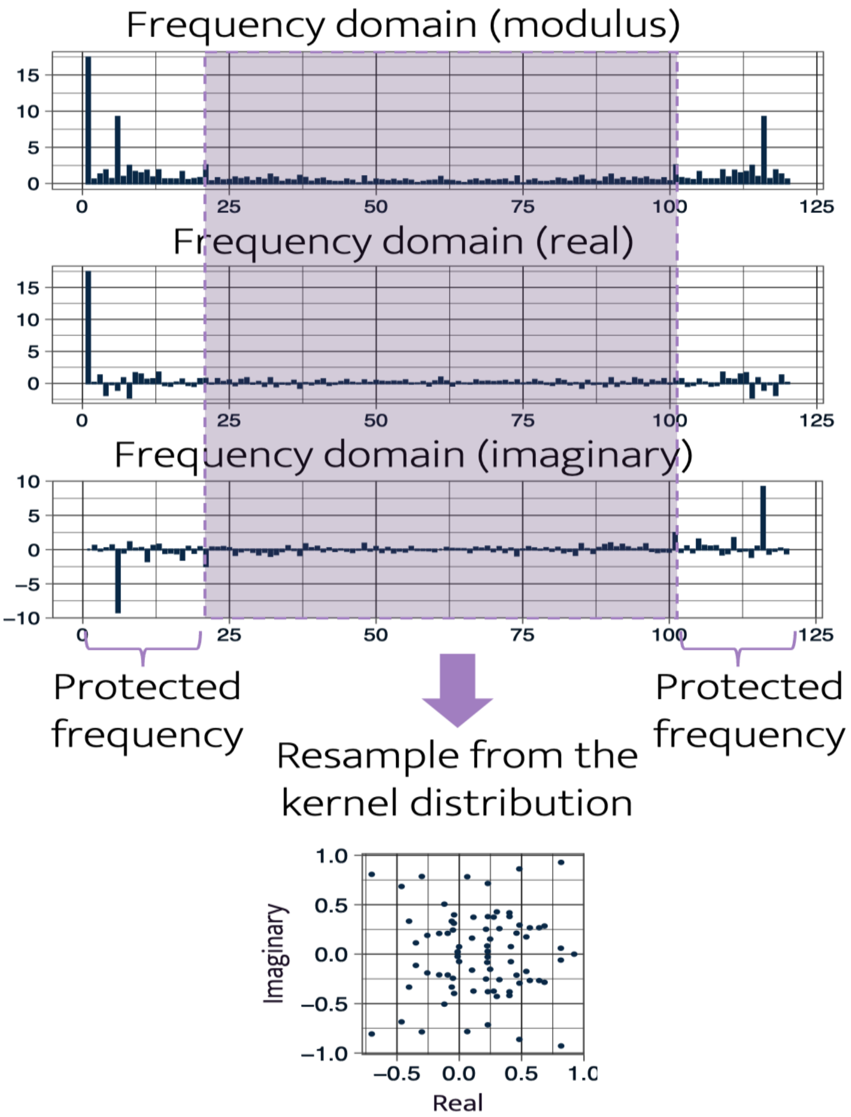

Supplementary Figure 2: DataSifter Longitudinal Obfuscation - DSLO Filtering Step.

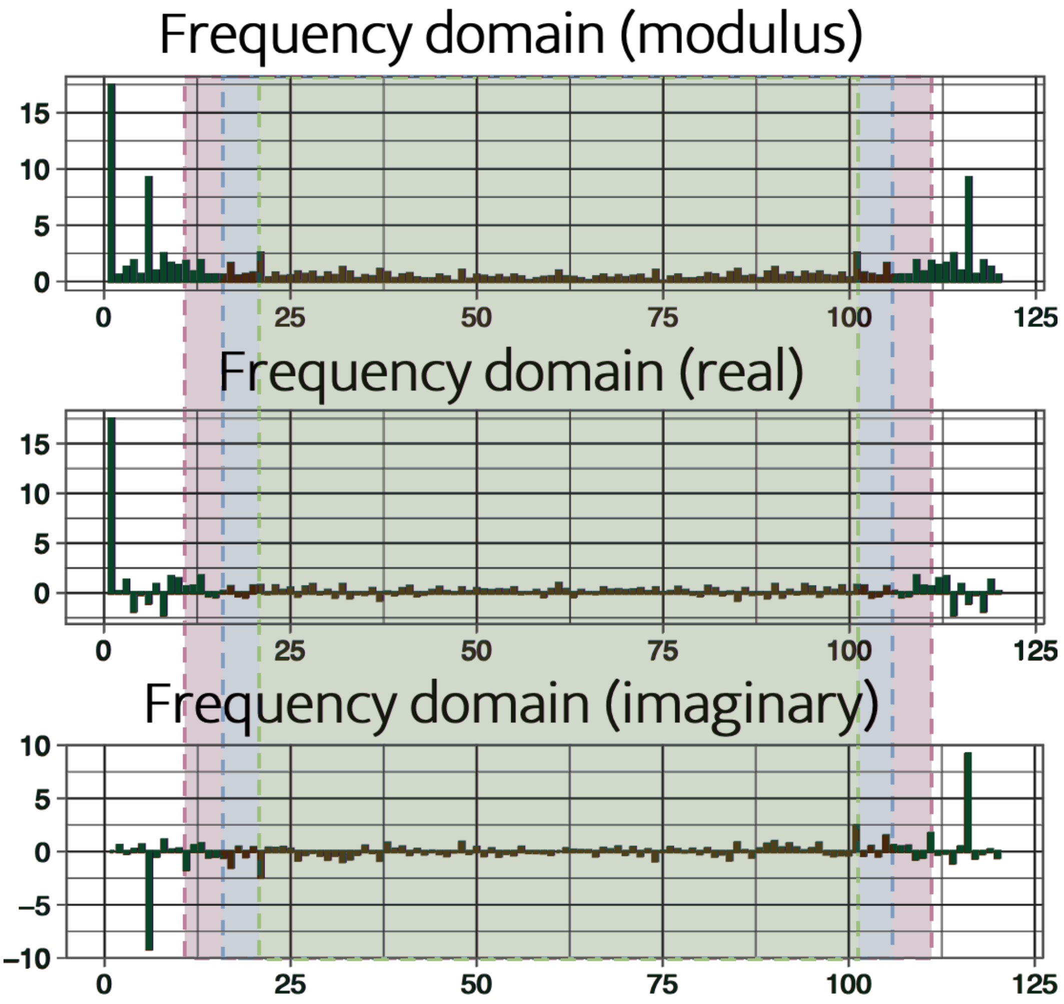

Supplementary Figure 3: DSLO different level of obfuscation. Different bandwidth ensures different levels of obfuscation. Each level is determined by pre-defined parameters, d and k, for three different levels of obfuscation. Namely 1/3 (small), 1/4 (medium) and 1/6 (large) for d and 1/2 (small), 1 (medium) and 2 (large). The different colors in the frequency plot are associated with the different values of d and k and different levels of obfuscation, namely low (green), medium (blue), and high (red).

The obfuscated time series in the frequency domain is back transformed into the time domain by inverse Fourier Transform. The transformed real part was retained, and the section

corresponding to missing values in the original time series was eliminated, which yielded the obfuscated data in the time domain (**Supplementary Figure 4**).

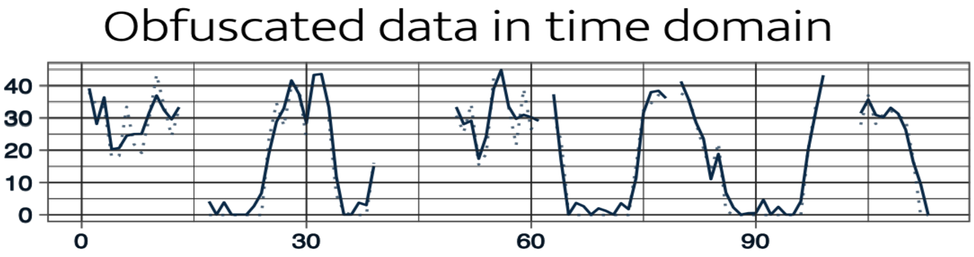

Supplementary Figure 4: DSLO transformation back in the time domain**.**

The obfuscated time series in the frequency domain is back transformed into the time domain by inverse Fourier Transform. The transformed real part was retained, and the section corresponding to missing values in the original time series was eliminated, which yielded the obfuscated data in the time domain.

DSLO does not allow, at this point, for multimodal data inputs; it deals with each time series independently for each subject. Given the scarcity of privacy and utility metrics for longitudinal data, by recasting the time series of AEB into daily averages (cross-sectional values) we were able to create this important variable from SDV methods and use the portfolio of metrics available through the SDmetrics library, including the ML utility analysis.

Supplementary Note 3 - Additional Synthetic Data Vault (SDV)

We describe ML algorithms used to generate synthetic data, using joint probability distributions using Gaussian copula and a *Conditional Tabular Generative Adversarial Network* (CTGAN).

A *Gaussian copula synthesizer* is a statistical method used to generate synthetic data by modeling dependencies between variables. It uses a copula, a function that links multivariate distributions to their marginal distributions, to capture the correlation structure between variables.[10] Gaussian copulas allow for the capturing of complex dependencies between variables, even when they are not normally distributed. In the Gaussian copula synthesizer, a multivariate normal distribution (Gaussian) is assumed for the copula, allowing for flexible modeling of dependencies, including nonlinear relationships. This approach is often used in financial modeling, risk management, and synthetic data generation to create datasets that preserve the relationships observed in the original data while allowing for privacy or scenario analysis. In the SDV package, the synthesizer first uses the cumulative distribution function (CDF) of the estimated marginal distributions to transform each variable to follow a uniform distribution. It then models the dependencies between the variables using a Gaussian (normal) distribution. Once this dependency structure is understood, the model can generate new synthetic data points that mimic the original data's relationships

CTGAN (Conditional Tabular Generative Adversarial Network) is a type of generative model specifically designed to generate realistic synthetic tabular data, and it was proposed by Lei Xu et al. in NeurIPS 2019.[11] It utilized the Conditional Generative Adversarial Network proposed by

Mehdi Mirza et al.[12] and made its performance exceed several widely-used Bayesian network baselines. While traditional GANs struggle with tabular data due to its mixed data types (categorical and continuous) and complex relationships, CTGAN addresses these challenges by using a conditional generator that conditions on discrete column values and a mode-specific normalization method to handle continuous columns. This allows CTGAN to effectively capture and replicate the underlying distributions and dependencies in the original dataset, making it a powerful tool for generating high-quality synthetic data for tasks such as data augmentation, privacy-preserving data sharing, and imbalanced dataset correction. In the SDV package, an end-to-end workflow for creating synthetic data called CTGAN Synthesizer is also provided. This workflow includes pre-processing the data and handling constraints, running the core machine learning algorithm (CTGAN) on the preprocessed data, and post-processing the synthetic data to meet the correct format and specifications.

The *PARSynthesizer* is a Probabilistic Auto-Regressive model that is based on neural networks. It learns how to create brand new sequences of multi-dimensional data, by conditioning on the unchanging, context values. uses a deep learning method to train a model and generate synthetic data.in general is less mature than the other SDV synthesizers we used in this work (e.g., CTGAN and GC). We decided not to use the PARSynthesizer for production runs because of the large CPU time to run each time series (~50-100 times slower than the DSLO), as well as because of its poor performance.

Supplementary Figures 5 and 6 show 2 ParticipantIDs time series plots as an example. The figures compare original vs synthetic data (DSLO and PARSynthesizer) and highlight the better performance of DSLO vs PARSynthesizer in replicating the original longitudinal data. The CPU computational timetable at the end of **Supplementary Note 4** also highlights the faster DSLO performance (~50-100 times faster).

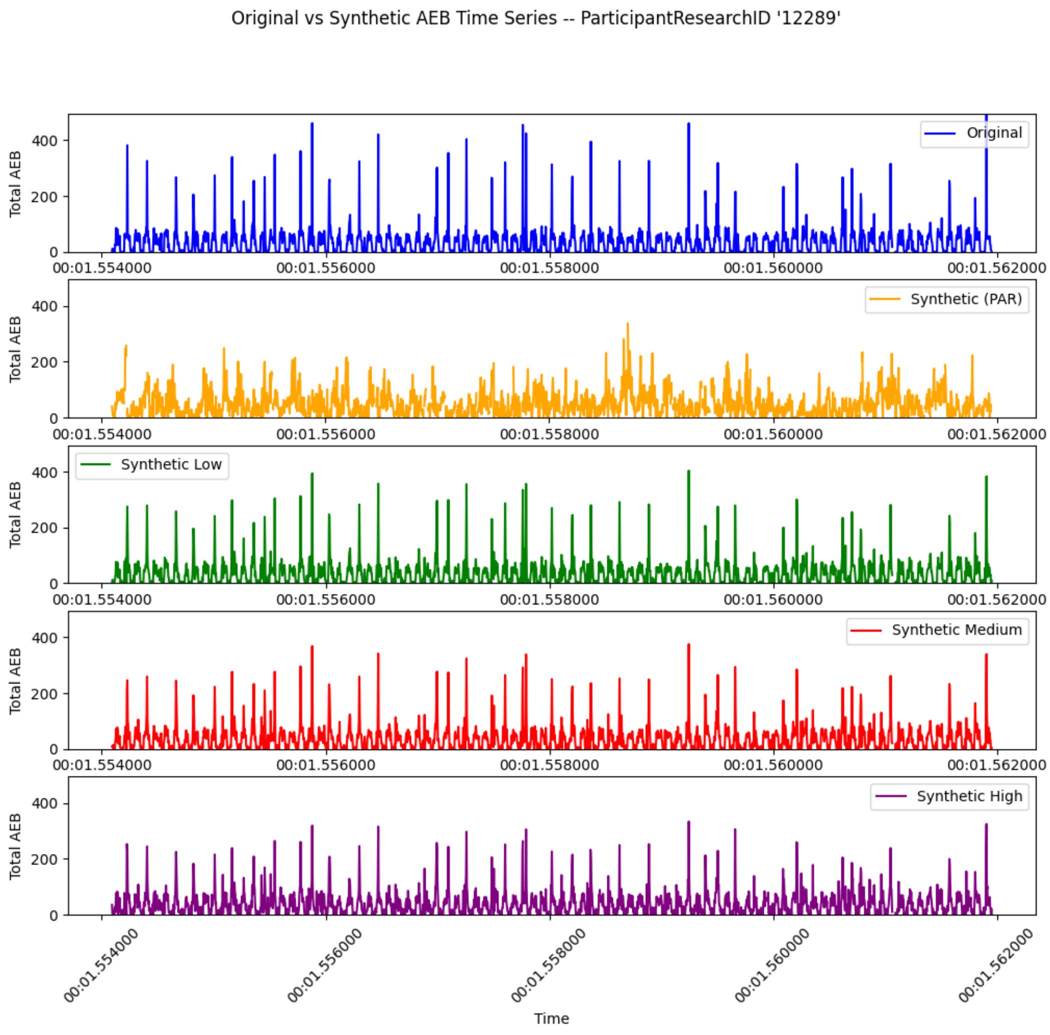

Supplementary Figure 5: Comparison of Original vs Synthetic iWatch longitudinal data for a single participant (ID 12289). DSLO synthetic time series (green, red, and purple) outperforms SDV-PARSynthesizers by capturing the spikes.

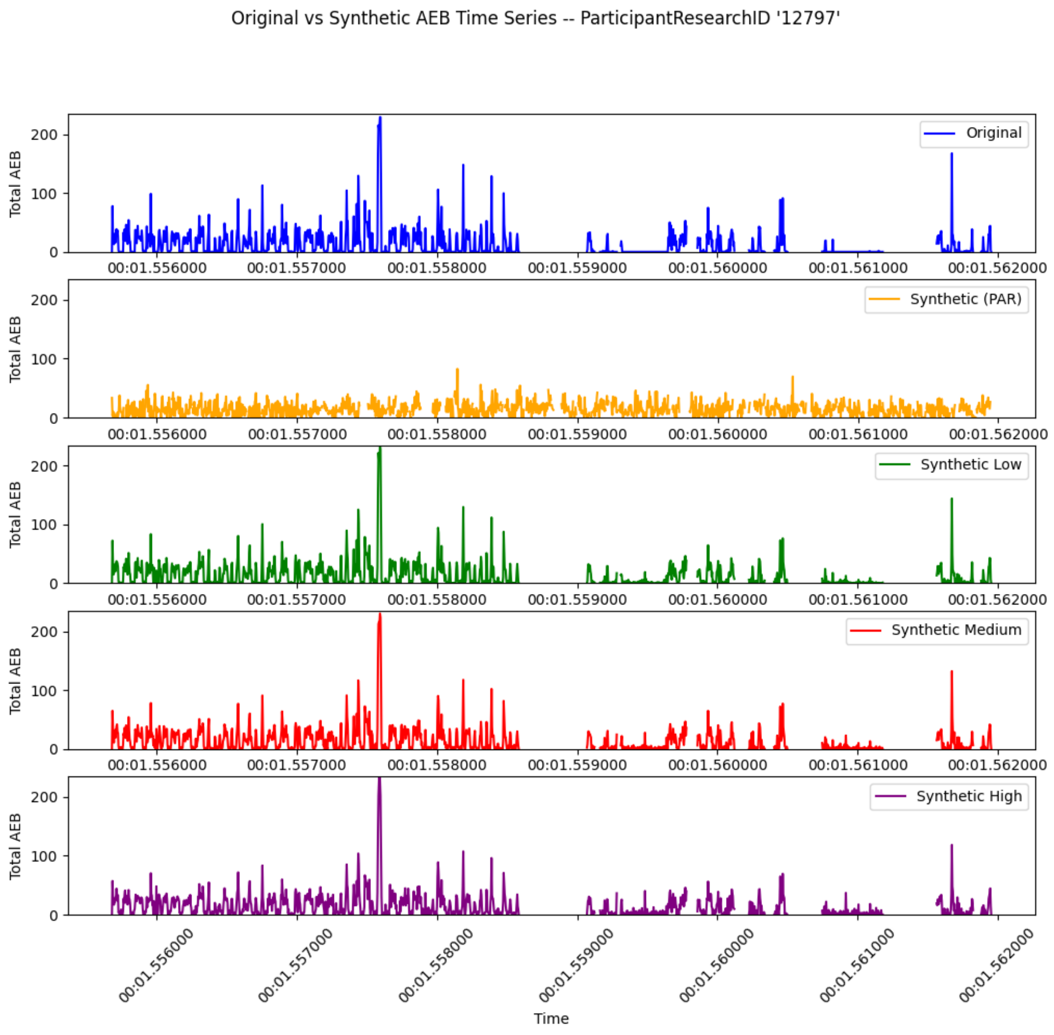

Supplementary Figure 6: Comparison of Original (blue) vs Synthetic iWatch longitudinal data for a single participant (ID 12797). DSLO synthetic time series (green, red, and purple) outperforms SDV-PARSynthesizers by capturing the spikes.

Supplementary Note 4 - Synthetic Data Evaluation Metrics

SDV privacy and utility metrics generation toolbox returns 2 main outputs by default from the SDMetrics library: a Quality Report and a Diagnostic Report. The Quality Report evaluates how well synthetic data captures mathematical properties from real data. This is also known as synthetic data fidelity. The report runs select metrics (e.g., column shapes and correlations) to measure these properties and summarizes the results. The score is normalized between 0 and 1, where 0 is usually not good, and 1 is best. The Diagnostic Report runs some basic checks to ensure that the synthetic data is usable and replicates some structural properties of the original data (column names, missingness, range of numerical and categorical variables, etc.). This can be referred to as the integrity check on the synthetic datasets. Again, the score is normalized between 0 and 1, where 0 is usually not good, and 1 is best. A larger set of metrics is also available. We classified them as either utility or privacy metrics.

Quality Report: Column Shapes Report

The shape of a column describes its overall distribution. The higher the score, the more similar the distributions of real and synthetic data. Column Shape is an average of 2 different metrics, KSComplement (numerical) and TVComplement (categorical).

1. *KSComplement*: Works on numerical and datetime columns. The KSComplement uses the Kolmogorov-Smirnov (KS) statistic. To compute this statistic, we convert a numerical distribution into its cumulative distribution function (CDF). The KS statistic is the maximum difference between the two CDFs. The distance is a value between 0 and 1. In SDMetrics, we invert the statistic: The KSComplement returns 1-(KS statistic) so that a higher score means higher quality. The score ranges from 0 (Worst: The real and

synthetic data are as different as they can be) to 1 (Best: The real data is exactly the same as the synthetic data).

2. *TVComplement*: Works on categorical and Boolean columns. It computes the Total Variation Distance (TVD) between the real and synthetic columns. The TVComplement returns 1 - TVD so that a higher score means higher quality. The score ranges from 0 (Worst: The real and synthetic data are as different as they can be) to 1 (Best: The real data is exactly the same as the synthetic data).

Variable	Metric	DS_small	DS_medium	DS_large	SDV_GC	SDV_CTGAN
AgeAtEnrollment	KSComplement	0.984153	0.8857709	0.7220205	0.9138329	0.9557610
EthnicityName	TVComplement	0.992739	0.9435457	0.8672829	0.9963684	0.8956751
GenderName	TVComplement	0.999006	0.9891053	0.8910532	0.9933972	0.9805216
Height_mean	KSComplement	0.992076	0.9240673	0.7603169	0.9607131	0.8511060
ICD10	KSComplement	0.723671	0.7834269	0.5117200	0.7702212	0.8544074
MaritalStatusName	TVComplement	0.997028	0.9577418	0.9148234	0.9858039	0.9138329
PreferredLanguage Name	TVComplement	0.998679	0.9937273	0.9838230	0.9996699	0.9243975
RaceName	TVComplement	0.997028	0.9861340	0.9144932	0.9798613	0.8398811
Sum_AEB	KSComplement	0.971937	0.9620337	0.9461869	0.8867613	0.9115220
Sum_Frequency	KSComplement	0.963024	0.9531198	0.9323209	0.9303400	0.9415649
Sum_hours	KSComplement	0.982502	0.9768901	0.9679762	0.9306702	0.8808188
Weight_mean	KSComplement	0.990095	0.9012876	0.7226808	0.9818422	0.8851106

Supplementary Table 1: Column Shapes Metrics Comparison.* The trend between two columns describes how they vary in relation to each other, for example contingency similarity

(categorical) or correlation similarity (numerical). The higher the score, the more the trends are alike.

Quality Report: Column Pair Trends Report

The trend between two columns describes how they vary in relation to each other, for example the correlation. The higher the score, the more the trends are alike.

CorrelationSimilarity: Works on numerical and datetime columns. For a pair of columns, A and B, this test computes a correlation coefficient on the real and synthetic data, R and S. This yields two separate correlation values. The test normalizes and returns a similarity score using the formula below:

$$score = 1 - \frac{\left|S_{A,B} - R_{A,B}\right|}{2}$$

Note that there are multiple ways to compute the correlation coefficient. This supports both the Pearson correlation coefficient and the Spearman's rank correlation coefficient. Both are bounded between -1 and +1. The score ranges from 0.0 (Worst: The pairwise correlations are as different as they can possibly be) to 1.0 (Best: The pairwise correlations of the real and synthetic data are exactly the same).

ContingencySimilarity: Works on categorical and Boolean columns. For a pair of columns, A and B, the test computes a normalized contingency table for the real and synthetic data. This table describes the proportion of rows that have each combination of categories in A and B. Then, it computes the difference between the contingency tables using the Total Variation Distance. Finally, we subtract the distance from *1* to ensure that a high score means high similarity. The process is summarized by the formula below.

$$score = 1 - \frac{1}{2}\sum_{\alpha \in A} \sum_{\beta \in B} \left|S_{\alpha,\beta} - R_{\alpha,\beta}\right|$$

In the formula, α describes all the possible categories in column A and β describes all the possible categories in column B. Meanwhile, R and S refer to the real and synthetic frequencies for those categories.The score ranges from 0.0 (Worst: The pairwise correlations are as different as they can possibly be) to 1.0 (Best: The pairwise correlations of the real and synthetic data are exactly the same). **Supplementary Table 2** shows both metrics for all the appropriate variables.

Variables		Metric	DS small	DS medium	DS large	SDV GC	SDV CTGAN
Age At Enrollment	Ethnicity Name	Contingency Similarity	0.97	0.80	0.49	0.81	0.86
Age At Enrollment	Gender Name	Contingency Similarity	0.97	0.80	0.54	0.83	0.89
Age At Enrollment	Height mean	Correlation Similarity	1.00	1.00	0.94	1.00	0.91
Age At Enrollment	ICD10	Correlation Similarity	1.00	0.99	0.92	1.00	0.93
Age At Enrollment	Marital Status Name	Contingency Similarity	0.97	0.76	0.53	0.77	0.80
Age At Enrollment	Preferred Language Name	Contingency Similarity	0.97	0.80	0.53	0.83	0.89
Age At Enrollment	Race Name	Contingency Similarity	0.97	0.78	0.45	0.79	0.81
Age At Enrollment	AVG AEB	Correlation Similarity	1.00	0.99	0.98	0.99	0.96

Age At Enrollment	Sum Frequency	Correlation Similarity	1.00	0.98	0.99	0.99	1.00
Age At Enrollment	Sum hours	Correlation Similarity	1.00	0.99	1.00	0.99	0.98
Age At Enrollment	Weight_mean	Correlation Similarity	1.00	0.99	0.92	0.98	0.96
Ethnicity Name	Height_mean	Contingency Similarity	0.99	0.87	0.64	0.87	0.77
Ethnicity Name	ICD10	Contingency Similarity	0.67	0.69	0.43	0.75	0.84
Ethnicity Name	Preferred Language Name	Contingency Similarity	0.99	0.94	0.86	0.99	0.84
Ethnicity Name	AVG AEB	Contingency Similarity	0.95	0.89	0.82	0.83	0.86
Ethnicity Name	Sum Frequency	Contingency Similarity	0.98	0.94	0.87	0.72	0.86
Ethnicity Name	Sum hours	Contingency Similarity	0.99	0.94	0.87	0.83	0.49
Ethnicity Name	Weight_mean	Contingency Similarity	0.98	0.84	0.65	0.84	0.83
Gender Name	Ethnicity Name	Contingency Similarity	0.99	0.94	0.81	0.99	0.90
Gender Name	Height_mean	Contingency Similarity	0.98	0.75	0.70	0.77	0.67
Gender Name	ICD10	Contingency Similarity	0.67	0.72	0.43	0.75	0.85

Gender Name	Marital Status Name	Contingency Similarity	1.00	0.96	0.86	0.98	0.91
Gender Name	Preferred Language Name	Contingency Similarity	1.00	0.99	0.88	0.99	0.92
Gender Name	Race Name	Contingency Similarity	0.99	0.94	0.87	0.96	0.84
Gender Name	AVG AEB	Contingency Similarity	0.95	0.93	0.87	0.84	0.92
Gender Name	Sum Frequency	Contingency Similarity	0.99	0.98	0.89	0.72	0.95
Gender Name	Sum hours	Contingency Similarity	0.99	0.98	0.89	0.84	0.50
Gender Name	Weight_mean	Contingency Similarity	0.98	0.84	0.69	0.83	0.86
Height_mean	ICD10	Correlation Similarity	1.00	0.99	0.99	0.99	0.98
Height_mean	Weight_mean	Correlation Similarity	1.00	0.81	0.92	0.98	0.84
Marital Status Name	Ethnicity Name	Contingency Similarity	0.99	0.93	0.82	0.97	0.87
Marital Status Name	Height_mean	Contingency Similarity	0.99	0.88	0.68	0.88	0.79
Marital Status Name	ICD10	Contingency Similarity	0.67	0.71	0.43	0.75	0.84
Marital Status Name	Preferred Language Name	Contingency Similarity	1.00	0.95	0.91	0.99	0.89

Marital Status Name	Race Name	Contingency Similarity	0.99	0.93	0.85	0.92	0.83
Marital Status Name	AVG AEB	Contingency Similarity	0.95	0.92	0.85	0.84	0.88
Marital Status Name	Sum Frequency	Contingency Similarity	0.99	0.94	0.88	0.72	0.89
Marital Status Name	Sum hours	Contingency Similarity	0.99	0.95	0.89	0.84	0.50
Marital Status Name	Weight_mean	Contingency Similarity	0.99	0.84	0.67	0.85	0.86
Preferred Language Name	Height_mean	Contingency Similarity	0.99	0.90	0.71	0.88	0.78
Preferred Language Name	ICD10	Contingency Similarity	0.67	0.73	0.43	0.75	0.84
Preferred Language Name	AVG AEB	Contingency Similarity	0.95	0.93	0.90	0.84	0.87
Preferred Language Name	Sum Frequency	Contingency Similarity	0.99	0.97	0.95	0.72	0.88
Preferred Language Name	Sum hours	Contingency Similarity	0.99	0.98	0.98	0.84	0.48
Preferred Language Name	Weight_mean	Contingency Similarity	0.99	0.85	0.69	0.84	0.84

Race Name	Ethnicity Name	Contingency Similarity	0.99	0.91	0.82	0.89	0.78
Race Name	Height_mean	Contingency Similarity	0.98	0.88	0.66	0.87	0.72
Race Name	ICD10	Contingency Similarity	0.67	0.71	0.43	0.75	0.80
Race Name	Preferred Language Name	Contingency Similarity	1.00	0.98	0.91	0.98	0.80
Race Name	AVG AEB	Contingency Similarity	0.95	0.92	0.86	0.83	0.80
Race Name	Sum Frequency	Contingency Similarity	0.98	0.96	0.90	0.72	0.82
Race Name	Sum hours	Contingency Similarity	0.98	0.96	0.90	0.82	0.47
Race Name	Weight_mean	Contingency Similarity	0.98	0.82	0.65	0.80	0.76
AVG AEB	Height_mean	Correlation Similarity	1.00	0.92	0.95	0.97	1.00
AVG AEB	ICD10	Correlation Similarity	1.00	0.99	0.98	0.98	0.99
AVG AEB	Sum Frequency	Correlation Similarity	1.00	1.00	1.00	0.98	0.62
AVG AEB	Sum hours	Correlation Similarity	0.99	0.99	0.98	1.00	0.63
AVG AEB	Weight_mean	Correlation Similarity	0.99	0.99	0.95	0.97	0.99

Sum Frequency	Height_mean	Correlation Similarity	1.00	1.00	0.97	1.00	0.97
Sum Frequency	ICD10	Correlation Similarity	1.00	0.99	0.98	0.98	0.96
Sum Frequency	Sum hours	Correlation Similarity	0.99	0.99	0.98	0.96	0.63
Sum Frequency	Weight_mean	Correlation Similarity	1.00	0.98	0.93	0.98	0.89
Sum hours	Height_mean	Correlation Similarity	1.00	1.00	0.97	1.00	0.96
Sum hours	ICD10	Correlation Similarity	1.00	0.99	0.97	0.99	0.97
Sum hours	Weight_mean	Correlation Similarity	1.00	0.99	0.95	0.99	0.99
Weight_mean	ICD10	Correlation Similarity	1.00	0.98	1.00	1.00	0.98

Supplementary Table 2: Column Pair Trends Metrics for Five Levels of Obfuscated Datasets

Diagnostic Report: Data Validity Report

The Diagnostic Report runs some basic checks to ensure that the synthetic data is usable. This report can be used as a first step to ensuring that we have created valid synthetic data. These metrics check if each column in the data contains valid data. **Supplementary Table 3** below shows all the metrics above for all the appropriate variables.

1. *KeyUniqueness*: Works on ID columns, usually the primary keys. This metric measures how many values in the synthetic data, S, are duplicates, meaning that there is another

value that is exactly the same. The score is the proportion of values that are *not* duplicates. The score ranges from 0 (Worst: None of the key values in the synthetic data are unique) to 1 (Best: All the key values in the synthetic data are unique).

2. *BoundaryAdherence*: Works on numerical, datetime columns. This metric computes the min and max values of the real column. Then, it computes the frequency of synthetic values that are in the [min, max] range. The score ranges from 0 (Worst: No value in the synthetic data is in between the min and max value of the real data) to 1 (Best: All values in the synthetic data respect the min/max boundaries of the real data).
3. *CategoryAdherence*: Works on categorical, Boolean columns. This metric extracts the set of unique categories that are present in the real column, C. Then it finds the data points of the synthetic data, S, that are found in the set C. The score is the proportion of these data points as compared to all the synthetic data points. The score ranges from 0 (Worst: None of the category values in the synthetic data were present in the real data) to 1 (Best: All category values in the synthetic data were present in the real data).

**Column**	**Metric**	**DS_small**	**DS_medium**	**DS_large**	**SDV_GC**	**SDV_CTGAN**
AgeAt Enrollment	Boundary Adherence	1	1	1	1	1
Gender Name	Category Adherence	1	1	1	1	1
MaritalStatus Name	Category Adherence	1	1	1	1	1
RaceName	Category Adherence	1	1	1	1	1

EthnicityName	Category Adherence	1	1	1	1	1
PreferredLanguageName	Category Adherence	1	1	1	1	1
Sum_AEB	Boundary Adherence	0.999339	0.9986794	0.998019	1	1
Sum Frequency	Boundary Adherence	0.9993397	0.9993397	0.999339	1	1
Sum_hours	Boundary Adherence	0.999669	0.9996699	0.999339	1	1
Height_mean	Boundary Adherence	1	1	1	1	1
Weight_mean	Boundary Adherence	1	1	1	1	1
ICD10	Boundary Adherence	0.717728	0.8342687	0.981181	0.829646	0.6130736

Supplementary Table 3: Data Validity Metrics for Five Levels of Obfuscated Datasets

Diagnostic Report: Data Structure Report

Supplementary Table 4 shows this metric measures whether the synthetic data captures the same table structure as the real data. We expect the synthetic data to have the same column names as the real data. *TableStructure*: This metric identifies all the column names in the real data and the synthetic data. The final score is based on the overlap between the columns of these datasets. The score ranges from 0 (Worst: There is no overlap in columns between the

real and synthetic data) to 1 (Best: The synthetic data has the same column names as the real data).

Metric	DS_small	DS_medium	DS_large	SDV_GC	SDV_CTGAN
TableStructure	1	1	1	1	1

Supplementary Table 4: Data Structure Metrics for Five Levels of Obfuscated Datasets

Overall Data Validity and Data Structure for both categorical and numerical variables.

For categorical or Boolean type features, the Overall Data Validity and Data Structure metric calculates the average score from the values of several basic functions, such as`CategoryAdherence`, `CategoryCoverage`, `TVComplement`, `MissingValueSimilarity`, `CSTest`. **Supplementary Table 5** shows the average and the single scores for GenderName, MaritalStatus, Ethnicity Name and Race Name.

Synthetic datasets	Overall	Gender Name	Marital Status Name	Ethnicity Name	Race Name
DS_small	0.99920768	0.999484	0.9993939	0.9985468	0.9994057
DS_medium	0.99267013	0.994327	0.9906281	0.9884977	0.9972268
DS_large	0.96845225	0.943553	0.9764274	0.9709299	0.9828986
SDV_GC	0.9972202	0.996562	0.9970730	0.9992733	0.9959723
SDV_CTGAN	0.97849798	0.989860	0.9797977	0.9763635	0.9679707

Supplementary Table 5: Results of the Overall Data Validity and Data Structure Scores on Categorical Features.

For numerical or datetime variable types, the Overall Data Validity and Data Structure metric calculates the average score of `BoundaryAdherence`, `RangeCoverage`, `KSComplement`, `MissingValueSimilarity`, `StatisticsSimilarity` (see *the Diagnostic Report* in the **Supplementary Note 4** for details on each metric results). **Supplementary Table 6** shows the average and the single scores for AgeAt Enrollment, Sum AEB, Sum Frequency and ICD10.

Synthetic datasets	Overall	Age At Enrollment	Sum AEB	Sum Frequency	ICD10
DS_small	0.966889	0.9967751	0.9927800	0.9912490	0.8867519
DS_medium	0.96870995	0.9738508	0.9900759	0.9888174	0.9220957
DS_large	0.94829598	0.9289793	0.9855730	0.9836932	0.8949384
SDV_GC	0.93212948	0.9792575	0.9401156	0.9071559	0.9019889
SDV_CTGAN	0.94537608	0.9900125	0.9790489	0.9785497	0.8338932

Supplementary Table 6: Results of the Overall Data Validity and Data Structure Scores on Numerical Features.

Privacy Metrics

A set of *privacy metrics* measures the risk of disclosing sensitive information through an inference attack. We used several ML algorithms for different sensitive target classes. The CategoricalCAP metrics works on categorical and Boolean data. The assumption is that some values in the real data are public knowledge. An attacker is combining this with synthetic data to make guesses about other real values that are sensitive. This metric describes how difficult it is for an attacker to correctly guess the sensitive information using an algorithm called Correct Attribution Probability (CAP).[18] CategoricalZeroCAP and CategoricalGeneralizedCAP are slight variations on the CAP algorithm that deal with missing values in the equivalence class in slightly different ways.[18] Privacy Against Inference Metrics are similar to CategoricalCAP. Privacy

Against Inference describes a set of metrics that calculate the risk of an attacker being able to infer real, sensitive values from the digital twin. Assume that a malicious attacker already possesses a few participant characteristics, captured in the information/columns of real dataset. In this case, the attacker may combine the prior information with the synthetic dataset and try to match meta-data to re-identify specific participants. In general, attackers can use sophisticated algorithms to match known metadata for some participants and information contained in the synthetically obfuscated (desensitized) digital twin set. These metrics vary depending on the data type of the target, namely numerical (i.e., NumericalMLP , NumericalLR and NumericalSVR ML algorithms are used[16,17,19] or categorical (CategoricalKNN, CategoricalNB, CategoricalRF and CategoricalEnsemble.[20–22] Detection Metrics use *LogisticDetection*[13,23] and *SVCDetection*[23,24] to calculate how difficult it is to tell apart the real data from the synthetic data.

The *CategoryCoverage* metric measures whether a synthetic column covers all the possible categories that are present in a real column. It first computes the number of unique categories, c, that are present in the real column r. Then it computes the number of those categories present in the synthetic column, s. It returns the proportion of real categories that are in the synthetic data, or

$$score = \frac{c_s}{c_r}.$$

The score ranges from *0.0* (Worst: suggesting that the synthetic column contains none of the categories present in the real column) to *1.0* (Best: where the synthetic column contains all the unique categories present in the real column). This *MissingValueSimilarity* metric compares whether the synthetic data has the same proportion of missing values as the real data for a given column. The score ranges from *0.0* (Worst: The synthetic data has a completely different proportion of missing values than the real data) to *1.0* (Best: The synthetic data perfectly

captures the proportion of missing values). The *NewRowSynthesis* metric measures whether each row in the synthetic data is novel, or whether it exactly matches an original row in the real data. It looks for matching rows between the real and synthetic dataset. To be considered a match, all the individual values in the real row must match the synthetic row.
The score ranges from *0.0* (Worst: All the rows in the synthetic data are copies of rows in the real data) to *1.0* (Best: The rows in the synthetic data are all new. There are no matches with the real data). The *RangeCoverage* metric measures whether a synthetic column covers the full range of values that are present in a real column. The score ranges from *0.0* (Worst: The synthetic column does not overlap at all with the range of values in the real column) to *1.0* (Best: The synthetic column covers the range of values present in the real column). This metric works on a single column each time.

The *StatisticSimilarity* metric measures the similarity between a real column and a synthetic column by comparing a summary statistic. Supported summary statistics are: mean, median and standard deviation. This test computes the given statistical function, f, for the real data and synthetic columns, r and s. Then, the test normalizes the score by scaling and taking its complement. This create a score that falls within the [*0,1*] range, where a high value means high similarity.

$$score = 1 - \frac{f(r) - f(s)}{max(r) - min(r)}$$

The score ranges from *0.0* (Worst: The statistic for the real data is extremely different from the synthetic data) to *1.0* (Best: The statistic evaluated on the real data is exactly the same as its counterpart computed on the synthetic data). This metric works one column at a time.
The *CSTest* metric computes the similarity of a real column vs. a synthetic column in terms of the column shapes. This test normalizes the real and synthetic data in order to compute the category frequencies. Then, it applies the Chi-squared test to test the null hypothesis that the

synthetic data comes from the same distribution as the real data. The test returns the p-value, where a smaller p-value indicates that the synthetic data is significantly different from the real data, rejecting the null hypothesis and leading to a worse overall score. The score ranges from *0.0* (Worst: The p-value is low, indicating that the synthetic data is significantly different than the real data) to *1.0* (Best: The p-value is high, indicating that the synthetic data is not very different from the real data). This metric also works on a single column at a time.

Data Likelihood describes a set of metrics that calculate the likelihood of the synthetic data belonging to the real data. Here we examine GMLogLikelihood (it fits multiple Gaussian mixture models to learn the distribution of the real data.[25,26] The model learns to produce a likelihood estimate for every row ranging from $-\infty$ to to ∞, where $-\infty$ means the row is likely not part of the data and ∞ means that it is. We apply the model to all the synthetic data and return the average likelihood score. The score ranges from $-\infty$ (Lowest: According to the algorithm used, the synthetic data has the lowest possible likelihood of belonging to the real data) to ∞ (Highest: According to the algorithm, the synthetic data has the highest possible likelihood of belonging to the real data).

The next set of results show metrics summarizing the risk of disclosing sensitive information through an inference attack. The scores range from 0 (Worst: The real data is not at all safe from the attack. The attacker can correctly guess every sensitive value by applying the chosen attack algorithm.) to 1 (Best: The real data is 100% safe from the attack. The attacker is not able to correctly guess any of the sensitive values by applying the chosen attack algorithm). These metrics vary depending on the data type of the target, namely categorical (**Supplementary Tables 7-9**) or numerical (**Supplementary Table 10**).

Supplementary Tables 7 and **8** show an example of Categorical Correct Attribution Probability (CAP) Metrics Comparison between Obfuscated Datasets with key fields being EthnicityName and GenderName, and sensitive field being RaceName and MaritalStatusName, respectively. **Supplementary Table 9** shows an example of evaluating a categorical feature as a sensitive field. **Supplementary Table 10** shows an example of evaluating a numerical feature as sensitive fields, AVG_AEB_day. **Supplementary Table 11** shows Detection Metrics results. Detection Metrics use *LogisticDetection*[28,29] and *SVCDetection*[29,30] to calculate how difficult it is to tell apart real data from synthetic data.

Synthetic datasets	Categorical CAP	Categorical Zero CAP	Categorical Generalized CAP
DS_small	0.6384908	0.6384908	0.6384908
DS_medium	0.6591422	0.6591422	0.6591422
DS_large	0.6361128	0.6361128	0.6361128
SDV_GC	0.6616735	0.6616735	0.6616735
SDV_CTGAN	0.7134487	0.7134487	0.7134487

Supplementary Table 7: *Categorical Correct Attribution Probability* (CAP) Metrics Comparison between 5 Levels of Obfuscated Datasets (*Key fields: EthnicityName and GenderName,Sensitive Field: RaceName)*

Synthetic datasets	Categorical CAP	Categorical Zero CAP	Categorical Generalized CAP
DS_small	0.6384908	0.6384908	0.6384908

Synthetic datasets	Categorical CAP	Categorical Zero CAP	Categorical Generalized CAP
DS_medium	0.6591422	0.6591422	0.6591422
DS_large	0.6361128	0.6361128	0.6361128
SDV_GC	0.6616735	0.6616735	0.6616735
SDV_CTGAN	0.7134487	0.7134487	0.7134487

Supplementary Table 8: *Categorical Correct Attribution Probability* (CAP) Metrics Comparison between 5 Levels of Obfuscated Datasets (*Key fields: EthnicityName and GenderName,Sensitive Field: MaritalStatusName)*

Synthetic datasets	Categorical KNN	Categorical NB	Categorical RF	Categorical Ensemble
DS_small	0.4638495	0.4546055	0.4546055	0.4546055
DS_medium	0.6021789	0.4546055	0.4546055	0.4546055
DS_large	0.4678112	0.4681413	0.4783757	0.4681413
SDV_GC	0.4717729	0.4638495	0.4638495	0.4638495
SDV_CTGAN	0.4711126	0.5146913	0.5156817	0.5146913

Supplementary Table 9: Categorical Privacy Against Inference Metrics Comparison between 5 Levels of Obfuscated Datasets (*Key fields: "EthnicityName", "GenderName", Sensitive_fields = "MaritalStatusName")*

Synthetic datasets	Numerical MLP	Numerical LR	Numerical SVR
DS_small	0.05996035	0.04936194	0.07489575
DS_medium	0.04820431	0.05171969	0.07532661
DS_large	0.06602836	0.07112810	0.07688653
SDV_GC	0.35511738	0.33563830	0.08571888
SDV_CTGAN	0.33047780	0.34818653	0.08179890

Supplementary Table 10: Numerical Privacy Against Inference Metrics Comparison between 5 Obfuscated Datasets (*Key fields: AgeAtEnrollment, Sum_Frequency, Height_mean, Weight_mean, and ICD10 (represented as A, B, C, D, E, F, G, H, I, J, K, L, M, N, O, Q, R, S, T, Z), Sensitive Field:* AVG_AEB_day*)*

Synthetic datasets	LogisticDetection	SVCDetection
DS_small	1.0000000	0.80361176
DS_medium	0.8773610	0.42247470
DS_large	0.6198796	0.05480052
SDV_GC	0.9014908	0.38876582
SDV_CTGAN	0.5900610	0.09325998

Supplementary Table 11: Detection Metrics Comparison between 5 Obfuscated Datasets *The score ranges from 0.0 (Worst: The machine learning model can perfectly identify synthetic data*

apart from the real data.) to 1.0 (Best: The machine learning model cannot identify the synthetic data apart from the real data).

CategoryCoverage, *MissingValueSimilarity*, *NewRowSynthesis, CSTest, Data Likelihood* and *RangeCoverage* metrics comparison across the five obfuscated datasets are shown in the Supplementary Material. **Supplementary Tables 12 and 13** below show *StatisticSimilarity* metrics across the five different obfuscated datasets for ICD10 and Sum_AEB

**Synthetic datasets**	**Statistic Similarity mean**	**Statistic Similarity median**	**Statistic Similarity std**
DS_small	0.9923597	0.9778870	0.9737124
DS_medium	0.9927831	0.8697789	0.9519784
DS_large	0.9817903	0.8230958	0.8104564
SDV_GC	0.8721101	0.9238329	0.7681863
SDV_CTGAN	0.9380363	0.9312039	0.9735784

Supplementary Table 12: Statistical Similarity *metric to evaluate the ICD10 column for the statistics mean, median, and standard deviation*

**Synthetic datasets**	**Statistic Similarity mean**	**Statistic Similarity median**	**Statistic Similarity std**
DS_small	0.9926225	0.9944955	0.9935941
DS_medium	0.9896667	0.9924329	0.9904359
DS_large	0.9836590	0.9880191	0.9840291

Synthetic datasets	Statistic Similarity mean	Statistic Similarity median	Statistic Similarity std
SDV_GC	0.9984796	0.9764257	0.9777268
SDV_CTGAN	0.9837226	0.9850150	0.9890950

Supplementary Table 13: Statistical Similarity *metric to evaluate the Sum_AEB column for the statistics mean, median, and standard deviation*

Utility Metrics

A set of *utility metrics* perform predictive analytics using several machine learning (ML) algorithms to classify a target/sensitive variable on the synthetic datasets and compare the prediction to the original dataset. These metrics allow for binary, multinomial and numerical outcomes to be predicted. They are referred to as ML efficacy metrics. The binary classification ML algorithms are BinaryAdaBoostClassifier, BinaryDecisionTreeClassifier, BinaryLogisticRegression, and BinaryMLPClassifier.[13–16] The multinomial classification ML algorithms are the MulticlassDecisionTreeClassifier and the MulticlassMLPClassifier.[16] The numerical/regression ML algorithms is Linear Regression.[17]

Regression metrics calculate the success of using synthetic data to perform an ML regression task. It returns the R^2 test score. It ranging in the interval from [*0,1*]. The lower limit, *0*, corresponds to the worst performance and suggesting that given training data a specific ML algorithm would not be expected to predict any of the test data correctly. Whereas the upper limit (*1.0*) corresponds to the optimal performance, which suggests that given a training dataset, a specific ML algorithm would be expected to perform its tasks with near perfect accuracy on prospective testing data.

We choose different target variables to generate the appropriate utility scores or machine learning efficacy metrics. The results should be interpreted as how accurately the ML model can predict the target variable, given the synthetic data as the training data and the testing data as the original data. GenderName (binary, **Supplementary Table 14**), MaritalStatusName (multinomial, **Supplementary Table 15**), EthnicityName (multinomial, **Supplementary Table 16**) and AVG_AEB_day (numerical, **Supplementary Table 17**) were chosen to include different data types as target outcomes (i.e., binary, multinomial and numerical). The scores returned by the binary and multinomial classification range from *0.0* (Worst: Given the training data with the provided ML algorithm, you will not be able to predict any of the test data correctly) to *1.0* (Best: Given the training data with the provided ML algorithm, you will be able to perform ML tasks with 100% accuracy on the test data predicting the target variable).

Synthetic datasets	BinaryAdaBoost Classifier	BinaryDecisionTree Classifier	BinaryLogistic Regression	BinaryMLP Classifier
DS_small	0.8401893	0.9181752	0.8381924	0.8514560
DS_medium	0.6532166	0.6332454	0.6947133	0.6830933
DS_large	0.5879608	0.4641109	0.7867510	0.6096512
SDV_GC	0.7975967	0.6816280	0.8087846	0.7849505
SDV_CTGAN	0.7342144	0.6255452	0.7620902	0.7768325

Supplementary Table 14: Machine Learning Binary Classification Metrics Comparison between 5 Levels of Obfuscated Datasets. Target: *GenderName*. This computes how accurately the ML model can predict the GenderName, given the synthetic_data as the training data and the testing data as the real data

Synthetic datasets	MulticlassDecision TreeClassifier	MulticlassMLP Classifier
DS_small	0.8121484	0.5440164
DS_medium	0.4992015	0.4856765
DS_large	0.3311335	0.3907030
SDV_GC	0.3833310	0.4437649
SDV_CTGAN	0.3752531	0.4217297

Supplementary Table 15: Machine Learning Multiclass Classification Metrics Comparison for `MaritalStatusName` between 5 Levels of Obfuscated Datasets

Synthetic datasets	MulticlassDecision TreeClassifier	MulticlassMLP Classifier
DS_small	0.7391466	0.4680676
DS_medium	0.2996822	0.2560228
DS_large	0.2437423	0.2290213
SDV_GC	0.2367344	0.2305296
SDV_CTGAN	0.2734490	0.2708184

Supplementary Table 16: Machine Learning Multiclass Classification Metrics Comparison for `EthinicityName` across 5 Obfuscated Datasets

Synthetic datasets	LinearRegression
DS_small	0.3826051
DS_medium	0.3520111
DS_large	0.1873403
SDV_GC	0
SDV_CTGAN	0

Supplementary Table 17: *Machine Learning Regression Metrics Comparison for `AVG_AEB_day'*

Consolidated Scores

We consolidated the metrics scores under a single score wherever feasible (e.g., similar ranges) and meaningful (e.g., similar interpretation). We compute the overall scores for privacy and utility metrics, respectively.

Overall Privacy Scores: If the column to be examined is categorical or Boolean type, then it calculates the average score of CategoryAdherence, CategoryCoverage, TVComplement, MissingValueSimilarity, CSTest. If the column to be examined is numerical or datetime type, then it calculates the average score of BoundaryAdherence, RangeCoverage, KSComplement, MissingValueSimilarity, StatisticsSimilarity. Some of the metrics examine the whole synthetic table. Thus the appropriate overall score calculates the average of NewRowSynthesis, LogisticsDetection, SVCDetection, and TableStructure. The score ranges from *0.0* (The synthetic data is not very different from the real data, or the machine learning model cannot identify the synthetic data apart from the real data) to *1.0* (The synthetic data is significantly different from the real data, the machine learning model can perfectly identify synthetic data apart from the real data).

Overall Utility Scores If the column to be examined is binary categorical or Boolean type, then it calculates the average score of BinaryAdaBoostClassifier, BinaryDecisionTreeClassifier, BinaryLogisticRegression, BinaryMLPClassifier. If the column to be examined is multiclass categorical or Boolean type, then it calculates the average score of MulticlassDecisionTreeClassifier, MulticlassMLPClassifier. If the column to be examined is numerical or datetime type, then this function will not provide an average utility score, since the metrics are not ranged from *0.0* to *1.0*.

Overall CategoricalCAP and Privacy against Inference Scores. The CategoricalCap Privacy against inference metric is appropriate only for categorical variable types and reflects the aggregate overall mean of a number of simpler metrics, including, CategoricalCAP, Categorical Zero CAP, Categorical Generalized CAP, Categorical KNN, Categorical NB, Categorical RF, and Categorical Ensemble (**Supplementary Table 18**).

We decided not to use the PARSynthesizer for production runs because of the large CPU time to run each time series (~50-100 times slower than the DSLO), as well as because of its poor performance (**Supplementary Table 20**).

**Synthetic datasets**	**GenderName**	**MaritalStatusName**	**EthnicityName**
DS_small	0.4630891	0.4587880	0.1644588
DS_medium	0.4703288	0.4945559	0.1814791
DS_large	0.4747112	0.4755979	0.1633389
SDV_GC	0.4736669	0.4960229	0.2213963
SDV_CTGAN	0.4790292	0.5091503	0.2061188

Supplementary Table 18: Overall CategoricalCAP and Privacy against Inference Scores for categorical values. It takes the average of **CategoricalCAP (Supplementary Tables 7-8), Categorical Zero CAP (Supplementary Tables 7-8), Categorical Generalized CAP (Supplementary Tables 7-8), Categorical KNN (Supplementary Table 9), Categorical NB (Supplementary Table 9), Categorical RF (Supplementary Table 9), and Categorical Ensemble (Supplementary Table 9), where Categorical Ensemble (Supplementary Table 9).** The scores range from 0 (Worst: The real data is not at all safe from the attack. The attacker can correctly guess every sensitive value by applying the chosen attack algorithm.) to 1 (Best:

The real data is 100% safe from the attack. The attacker is not able to correctly guess any of the sensitive values by applying the chosen attack algorithm).

The Numerical Privacy Against inference is appropriate only for numerical variable types and reflects the aggregate overall mean of a number of simpler metrics, including, Numerical MLP, Numerical LR and Numerical SVR (see **Supplementary Table 13**).

**Synthetic datasets**	**AVG_AEB_day**	**BMI_mean**	**ICD10**
DS_small	0.05892467	0.08923978	0.08704495
DS_medium	0.06724003	0.09238845	0.08488092
DS_large	0.07451116	0.11320772	0.08119381
SDV_GC	0.25348774	0.14732064	0.09850664
SDV_CTGAN	0.25586749	0.33114661	0.09530591

Supplementary Table 19: Overall Numerical Privacy against Inference Scores for numerical values. It takes the average of **Numerical MLP, Numerical LR, and Numerical SVR (Supplementary Table 10).** The scores range from 0 (Worst: The real data is not at all safe from the attack. The attacker can correctly guess every sensitive value by applying the chosen attack algorithm.) to 1 (Best: The real data is 100% safe from the attack. The attacker is not able to correctly guess any of the sensitive values by applying the chosen attack algorithm).

Synthetic data generation	CPU time
DataSifter	~20 minutes
SDV [GC and CTGAN]	~25 minutes
DSLO (1 time series)	<1 second
PArSynthesizer DSLO (1 time series)	~50-100 seconds

Privacy and Utility Metrics	CPU time [secs]
QualityReportGeneral	3.198130541
ColumnShapes&ColumnPairTrends	3.11781591997
DiagnosticReportGeneral	0.05986990033
DataValidity&DataStructure	0.05707124991
MLEfficacy-BinaryClassification	4.47432397999
MLEfficacy-MulticlassClassification`MaritalStatusName`	10.0060269999
MLEfficacy-MulticlassClassification`EthnicityName`	4.0468389999
MLEfficacy-Regression`ICD10`	3.53815770007
MLEfficacy-Regression`Sum_AEB`	3.4618700036
CategoricalCAP,key_fields EthnicityName,GenderName	66.641182333
Detection	37.255320959
GuessingNumericalValue,key_fields AgeAtEnrollment,Sum_AEB,Sum_Frequency,Sum_hours,Weight_mean, Height_mean	47.65681400
CategoryCoverage`EthnicityName`	0.03057999285

CategoryCoverage`GenderName`	0.02925070898
CategoryCoverage`MaritalStatusName`	0.02897716642
MissingValueSimilarity`EthnicityName`	0.02677028786
MissingValueSimilarity`GenderName`	0.02715699833
NewRowSynthesis	78.8480558001
RangeCoverage`ICD10`	0.02607537897
StatisticSimilarity`ICD10`	0.04424820965
StatisticSimilarity`Sum_AEB`	0.04747720697
CSTest`ICD10`	0.03859200134
DataLikelihood	35.9690909998
OverallPrivacywithColumn	0.08415898907
OverallPrivacywithoutColumn	116.089315
OverallUtilitytarget`Sum_AEB`	4.45331841991

Supplementary Table 20: Computational Complexity

Supplemental Information Bibliography

1. Little RJA. A test of missing completely at random for multivariate data with missing values. *J Am Stat Assoc*. 1988;83(404):1198-1202.

2. Stekhoven DJ, Bühlmann P. MissForest-non-parametric missing value imputation for mixed-type data. *Bioinformatics*. 2012;28(1):112-118.

3. Dinov I, Vandervest J, Marino S. *Electronic Medical Record Datasifter*. Google Patents; 2020.

4. Marino S, Zhou N, Zhao Y, Wang L, Wu Q, Dinov ID. HDDA: DataSifter: statistical obfuscation of electronic health records and other sensitive datasets. *J Stat Comput Simul*. 2018;89(2):249-271.

5. Zhou N, Wang L, Marino S, Zhao Y, Dinov ID. DataSifter II: Partially synthetic data sharing of sensitive information containing time-varying correlated observations. *J Algorithm Comput Technol*. 2022;16:174830262110653.

6. Apaydin H, Taghi Sattari M, Falsafian K, Prasad R. Artificial intelligence modelling integrated with Singular Spectral analysis and Seasonal-Trend decomposition using Loess approaches for streamflow predictions. *J Hydrol (Amst)*. 2021;600(126506):126506.

7. Cleveland RB, William S, Cleveland JE, Mcrae I. STL: A seasonal-trend decomposition. *J off Stat*. 1990;6(1):3-73.

8. Heckbert P. Fourier transforms and the fast Fourier transform (FFT) algorithm. Computer Graphics. 1995;2:15-463.

9. Węglarczyk S. Kernel density estimation and its application. *ITM Web Conf*. 2018;23:00037.

10. Li Z, Zhao Y, Fu J. SYNC: A copula based framework for generating synthetic data from aggregated sources. *arXiv [statAP]*. Published online September 20, 2020. http://arxiv.org/abs/2009.09471

11. Xu L, Skoularidou M, Cuesta-Infante A, Veeramachaneni K. Modeling Tabular data using Conditional GAN. *arXiv [csLG]*. Published online June 30, 2019. http://arxiv.org/abs/1907.00503

12. Mirza M, Osindero S. Conditional generative Adversarial Nets. *arXiv [csLG]*. Published online November 6, 2014. http://arxiv.org/abs/1411.1784

13. Klein M, Kleinbaum DG, Pryor ER. *Logistic Regression*. 2nd ed. Springer; 2002.

14. Li X, Wang L, Sung E. AdaBoost with SVM-based component classifiers. *Eng Appl Artif Intell*. 2008;21(5):785-795.

15. Swain PH, Hauska H. The decision tree classifier: Design and potential. *IEEE Trans Geosci Electron*. 1977;15(3):142-147.

16. Windeatt T. Ensemble MLP Classifier Design. In: *Studies in Computational Intelligence*. Studies in computational intelligence. Springer Berlin Heidelberg; 2008:133-147.

17. Weisberg S. *Applied Linear Regression*. 3rd ed. John Wiley & Sons; 2005. https://books.google.com/books?hl=en&lr=&id=xd0tNdFOOjcC&oi=fnd&pg=PR7&dq=Weisberg,+S.,+Applied+linear+regression.+Vol.+528.+2005:+John+Wiley+%26+Sons.&ots=dW3sBpJzMO&sig=1ZVrYduNx-YDkXkC4ntFmalJMK0

18. Taub J, Elliot M, Pampaka M, Smith D. Differential correct attribution probability for synthetic data: An exploration. In: *Privacy in Statistical Databases*. Lecture notes in computer science. Springer International Publishing; 2018:122-137.

19. Brereton RG, Lloyd GR. Support vector machines for classification and regression. *Analyst*. 2010;135(2):230-267.

20. Kramer O. *Dimensionality Reduction with Unsupervised Nearest Neighbors*. 2013th ed. Springer; 2013.

21. Rigatti SJ. Random Forest. *J Insur Med*. 2017;47(1):31-39.

22. Webb GI, Keogh E, Miikkulainen R. Encyclopedia of machine learning. 2010;15:713-714.

23. Elliot M. *Final Report on the Disclosure Risk Associated with the Synthetic Data Produced by the Sylls Team*.; 2015.

24. Zhang Y. Support vector machine classification algorithm and its application. In: *Communications in Computer and Information Science*. Communications in computer and information science. Springer Berlin Heidelberg; 2012:179-186.

25. Chokwitthaya C. *Applying the Gaussian Mixture Model to Generate Large Synthetic Data from a Small Data Set. in Construction Research Congress 2020*. American Society of Civil Engineers; 2020.

26. Reynolds D. Gaussian Mixture Models. In: *Encyclopedia of Biometrics*. Springer US; 2009:659-663.
